# Spasmolytic Activity and Anti-Inflammatory Effect of Novel Mebeverine Derivatives

**DOI:** 10.3390/biomedicines12102321

**Published:** 2024-10-12

**Authors:** Mihaela Stoyanova, Miglena Milusheva, Vera Gledacheva, Iliyana Stefanova, Mina Todorova, Nikoleta Kircheva, Silvia Angelova, Mina Pencheva, Kirila Stojnova, Slava Tsoneva, Stoyanka Nikolova

**Affiliations:** 1 Department of Organic Chemistry, Faculty of Chemistry, University of Plovdiv, 4000 Plovdiv, Bulgaria; stoyanova@uni-plovdiv.bg (M.S.); miglena.milusheva@uni-plovdiv.bg (M.M.); minatodorova@uni-plovdiv.bg (M.T.); 2 Department of Bioorganic Chemistry, Faculty of Pharmacy, Medical University of Plovdiv, 4002 Plovdiv, Bulgaria; 3 Department of Medical Physics and Biophysics, Faculty of Pharmacy, Medical University of Plovdiv, 4002 Plovdiv, Bulgaria; vera.gledacheva@mu-plovdiv.bg (V.G.); iliyana.stefanova@mu-plovdiv.bg (I.S.); mina.pencheva@mu-plovdiv.bg (M.P.); 4 Institute of Optical Materials and Technologies “Acad. J. Malinowski”, Bulgarian Academy of Sciences, 1113 Sofia, Bulgaria; nkircheva@iomt.bas.bg (N.K.); sea@iomt.bas.bg (S.A.); 5 University of Chemical Technology and Metallurgy, 8 St. Kliment Ohridski Blvd, 1756 Sofia, Bulgaria; 6 Department of General and Inorganic Chemistry with Methodology of Chemistry Education, Faculty of Chemistry, University of Plovdiv, 4000 Plovdiv, Bulgaria; stoynova@uni-plovdiv.bg; 7 Department of Analytical Chemistry and Computer Chemistry, University of Plovdiv, 4000 Plovdiv, Bulgaria; slava.tsoneva@uni-plovdiv.bg

**Keywords:** mebeverine, synthesis, in silico, ADMET, DFT, docking, spasmolytic activity, albumin denaturation, anti-inflammatory activity

## Abstract

**Background**: Irritable bowel syndrome (IBS) has a major negative influence on quality of life, causing cramps, stomach pain, bloating, constipation, etc. Antispasmodics have varying degrees of efficacy. Mebeverine, for example, works by controlling bowel movements and relaxing the muscles of the intestines but has side effects. Therefore, more efficient medication is required. **Methods**: In the current study, we investigated the synthesis of novel mebeverine analogs and determined ex vivo their spasmolytic and in vitro and ex vivo anti-inflammatory properties. The ability to influence both contractility and inflammation provides a dual-action approach, offering a comprehensive solution for the prevention and treatment of both conditions. **Results**: The results showed that all the compounds have better spasmolytic activity than mebeverine and good anti-inflammatory potential. Among the tested compounds, **3**, **4a**, and **4b** have been pointed out as the most active in all the studies conducted. To understand their mechanism of activity, molecular docking simulation was investigated. The docking analysis explained the biological activities with their calculated Gibbs energies and possibilities for binding both centers of albumin. Moreover, the calculations showed that molecules can bind also the two muscarinic receptors and interleukin-β, hence these structures would exert a positive therapeutic effect owed to interaction with these specific receptors/cytokine. **Conclusions**: Three of the tested compounds have emerged as the most active and effective in all the studies conducted. Future in vivo and preclinical experiments will contribute to the establishment of these novel mebeverine derivatives as potential drug candidates against inflammatory diseases in the gastrointestinal tract.

## 1. Introduction

Irritable bowel syndrome (IBS) has a major negative influence on quality of life [1,2]. Patients usually underestimate the influence of IBS on their life [3]. The quality of life of individuals with IBS is much lower than that of individuals with diabetes mellitus, dialysis-dependent end-stage renal disease, or individuals with acid reflux disease [4,5,6]. IBS is characterized by specific intestinal symptoms, varying in all patients. However, there are certain recurring patterns: cramps, frequent stomach pains, bloating and gas, constipation, diarrhea, or both, food intolerances, indigestion, chronic fatigue, and difficulty sleeping [7]. Along with changes in bowel habits, the primary clinical symptom of IBS is abdominal pain during bowel movements [8]. The following medical disorders can occasionally exist with IBS: a minor type of celiac disease that compromises the integrity of the intestines, excessive levels of serotonin in the colon, a slowing down of the excretory system, and severe cramping. Though the exact pathophysiology of IBS remains unclear, disruptions in the gut–brain axis, dysbiosis of the intestinal microbiota, aberrant motility of the gut, visceral hypersensitivity, and malfunction of the local immune system are all hypothesized to impact the development of the disease [9]. It is thought that either a sharp decrease in the immune system or a sharp rise in the colon’s sensitivity is what causes the illness. There are instances where post-infectious IBS is discussed, which is brought on by an infectious condition that has impacted the gut microbiota [10]. There are numerous potential causes for this illness, therefore it is challenging to pinpoint a single cause and to take preventative measures.

IBS is diagnosed mostly based on clinical symptoms; further testing is not usually advised. There is still no gold standard for diagnosing IBS, despite the development of numerous diagnostic criteria, such as Manning, Kruis, and Rome I–IV [11,12,13].

Treatment is still difficult and depends on the symptoms. The range of therapeutic options includes both pharmaceutical treatment and non-pharmacological management, such as food and lifestyle adjustments, contingent on the prevailing symptoms, such as diarrhea, constipation, or stomach pain [14]. Given that prior meta-analyses have shown some benefits over placebo, antispasmodics are presently the recommended treatment of choice for IBS patients experiencing stomach pain [15]. Different antispasmodic medications, however, have varying degrees of efficacy. Mebeverine, for example, is an antispasmodic used to treat intestinal smooth muscle spasms and intestinal functional abnormalities associated with IBS in order to relieve symptoms of abdominal pain [16]. It works by controlling bowel movements and relaxing the muscles of the intestines (Table 1). Consequently, low-grade mucosal inflammation and T-type calcium channel activity are linked to stomach pain [17]. In this case, the immune system considers vital food, cells lining the colon, and intestinal bacteria as intruders and attacks them. This causes inflammation and ulcers in the inner lining of the colon. In patients treated with mebeverine, a change in the serum levels of a number of biochemical markers was recorded, including the mediators and regulators of immunity and inflammation interleukins [18]. On the other hand, its systemic use typically results in adverse effects such as headaches, nausea, dizziness, and skin sensitivities (Table 1) [19,20].

Common painkillers, such as non-steroidal anti-inflammatory drugs (NSAIDs), acetaminophen, and aspirin, do not successfully relieve the symptoms of IBS. Anti-inflammatory drugs help the body control inflammation, but they do not address the underlying cause and have many gastrointestinal side effects, including bleeding and ulcers, which are especially dangerous for the elderly [21,22]. Inflammation is a vital part of body’s immune system and plays a protective role against tissue damage, microbial infection, etc. Acute inflammation is an essential part of innate immunity that removes harmful stimuli and promotes healing of affected tissues [23]. At the site of damage, pro-inflammatory cytokines and chemokines released by different immune cells aid in initiating the defense against injury [24]. Later, the body’s tissues and systemic homeostasis are restored by stimulating anti-inflammatory cytokine release. However, beneficial mechanisms may be inhibited or transform into harmful ones, which can lead to chronic inflammation that damages tissue. The intricate process of inflammation’s molecular mechanism involves proinflammatory cytokines including interleukin (IL)-1 and tumor necrosis factor (TNF)-α being released by activated T cells, monocytes, and macrophages [25].

The majority of drugs used to treat inflammation are NSAIDs, which not only effectively reduce inflammation but also have mild to moderate adverse effects. Diclofenac, for example, is a widely used medication for the management of acute inflammation and pain. It works by decreasing the levels of proinflammatory cytokines, vascular reactive oxygen species, and circulating C-reactive protein and inhibiting the release of IL-6, IL-1β, and TNF-α from monocytes and macrophages. Chen et al. found that inhibition of non-selective cation channels and membrane potential-dependent L-type Ca^2+^ channels led to a decrease in the cytosolic calcium level during isometric recording of smooth muscle contractile activity [26], and Callingham et al. postulated an effect on K^+^ channels [27]. These findings imply that diclofenac influences intracellular transduction mediated by SM receptors.

Therefore, in the current study, we investigated the synthesis of a number of novel mebeverine analogs and compared their biological characteristics to mebeverine and diclofenac.

## 2. Materials and Methods

All solvents and reagents were purchased from Merck (Merck KGaA, Darmstadt, Germany). Melting points were measured on a Kruss M5000 melting point meter (A.Krüss Optronic GmbH, Hamburg, Germany). All the compounds were characterized using ^1^H-NMR, ^13^C-NMR, IR, and HRESIMS. The purity of these compounds was determined by TLC. TLC was carried out on precoated 0.2 mm Fluka silica gel 60 plates (Merck KGaA, Darmstadt, Germany), using chloroform:diethyl ether:n-hexane = 6:3:1 as a chromatographic system. Neutral Al_2_O_3_ was used for column chromatographic separation. After evaporation of the solvent, the products were purified using recrystallization from diethyl ether. IR spectra were determined on a VERTEX 70 FT-IR spectrometer (Bruker Optics, Ettlingen, Germany). Spectra of ^1^H-NMR and ^13^C-NMR were recorded on a Bruker Avance III HD 500 spectrometer (Bruker, Billerica, MA, USA) at 500 MHz (^1^H-NMR) and 125 MHz (^13^C-NMR), respectively. Chemical shifts are given in relative ppm and were referenced to tetramethylsilane (TMS) (δ = 0.00 ppm) as an internal standard; the coupling constants are indicated in Hz. The NMR spectra were recorded at room temperature (ac. 295 K). HRESIMS spectra were acquired in positive mode on a Q Exactive Plus (ThermoFisher Scientific, Inc., Bremen, Germany) mass spectrometer, equipped with a heated HESI-II source. Operating conditions for the HESI source used in a positive ionization mode were +3.5 kV spray voltage, 320 °C capillary and probe heater temperature, a sheath gas flow rate of 36 AU, an auxiliary gas flow rate of 11 AU, a spare gas flow rate of 1 AU (AU refers to arbitrary values set by the Exactive Tune software 2.4) and an S-Lens RF level of 50.00. Nitrogen was used for sample nebulization and collision gas in the HCD cell. The aliquots of 1 µL of the solutions of the samples (ca. 20 µg mL^−1^) were introduced into the mass spectrometer via LC system Thermo Scientific Dionex Ultimate 3000 RSLC (Thermo Fisher Scientific, Germering, Germany) consisting of a 6-channel SRD-3600 degasser, HPG-3400RS high-pressure gradient pump, WPS-3000TRS autosampler, and TCC-3000RS column compartment equipped with narrow bore Hypersil GOLD™ C18 (2.1 × 50 mm, 1.9 μm) column. Each chromatographic run was carried out isocratically with a mobile phase consisting of water/acetonitrile/methanol/acetic acid (25:50:25:0.2). The solvent flow rate was 300 μL min^−1^. Full MS–SIM was used in an MS experiment in negative and positive mode, where the resolution, automatic gain control (AGC) target, maximum injection time (IT), and mass range were 70,000 (at *m*/*z* 200), 3 × 10^6^, 100 ms, and *m*/*z* 100–500, respectively. Xcalibur (Thermo Fisher Scientific, Waltham, MA, USA) ver. 4.0 was used for data acquisition and processing.

### 2.1. Synthesis of 2-amino-N-(1-(3,4-dimethoxyphenyl)propan-2-yl)benzamide ***3*** and Its Diamides ***4a***–***d***

A mixture of isatoic anhydride (1.63 g, 10 mmol) and 1-(3,4-dimethoxyphenyl)propan-2-amine (initially prepared from 1-(3,4-dimethoxyphenyl)propan-2-one) [28,29] (2.93 g, 15 mmol) dissolved in dichloromethane (30 mL) was stirred overnight at rt. The resulting solution was filtered on neutral Al_2_O_3_ and concentrated. Spectral data confirmed the structure of the obtained 2-amino-N-(1-(3,4-dimethoxyphenyl)propan-2-yl)benzamide (Supplementary Materials, Figures S1–S4).

2-amino-N-(1-(3,4-dimethoxyphenyl)propan-2-yl)benzamide (**3**): m.p. 152.8 °C, ^1^H NMR (500 MHz, CDCl_3_) δ 1.14 (d, *J* = 6.7, 3H, CH_3_), 2.70–2.80 (m, 2H, CH_2_), 3.75 (s, 3H, OCH_3_) 3.78 (s, 3H, OCH_3_), 4.32 (dq, *J* = 13.4, 6.7 Hz, 1H, CH), 5.51 (broad s, 1H, NH_2_), 5.86 (d, *J* = 7.9 Hz, 1H, NH), 6.56–6.59 (m, 1H, Ar), 6.66–6.68 (m, 3H, Ar), 6.74 (d, *J* = 5, 1H, Ar), 7.11–7.15 (m, 2H, Ar); ^13^C NMR (126 MHz, CDCl_3_) δ 168.49, 148.86, 147.72, 147.46, 132.25, 130.37, 126.98, 121.59, 117.91, 117.43, 117.07, 112.62, 111.18, 55.91, 55.84, 46.16, 41.66, 19.99; FT-IR, cm^−1^: 3467 ν_as_ (-N-H, -NH_2_), 3371 ν_s_ (-N-H, -NH_2_), 3283 ν (-N-H, >NH-amide), 3067, 3055, 3033 ν (Csp^2^-H, -Ph), 2998 ν_as_ (Csp^3^-H, -OCH_3_), 2972 ν_as_ (Csp^3^-H, -CH_3_), 2935, 2914 ν_as_ (Csp^3^-H, -CH_2_-), 2874 ν_s_ (Csp^3^-H, -CH_3_), 2838 ν_s_ (Csp^3^-H, -OCH_3_), ν_s_ (Csp^3^-H, -CH_2_-), 1626 ν (>C=O), secondary amide I, 1584 ν (C-C=C, -Ph_1,2,4_), 1539, 1514 δ(N-H) and ν (C-N), trans-secondary amide II, 1463 ν (C-C=C, -Ph_ortho_), ν (C-C=C, -Ph_1,2,4_), δ_as_ (-CH_3_), δ_as_(-OCH_3_), 1447 δ_s_ (-OCH_3_), 1416 ν (C-C=C, -Ph_1,2,4_), 1358 δ_s_ (-CH_3_), 1300 ν (C-C=C, -Ph_ortho_), ν (C-N), secondary amide III, 1256 δ (-Csp^2^-H, -Ph_1,2,4_), 1157 δ (-Csp^2^-H, -Ph_ortho_), ρ(-CH-)/ν (N-C) in –NH-CH-, 1139 δ (-Csp^2^-H, -Ph_1,2,4_), 1029 δ (-Csp^2^-H, -Ph_ortho_); HRMS electrospray ionization (ESI) *m*/*z* calcd. for [M + H]^+^ C_18_H_23_O_3_N_2_^+^ = 315.17032, found 315.16956 (mass error ∆m = −2.41 ppm).

To a solution of 3 mmol 2-amino-N-(1-(3,4-dimethoxyphenyl)propan-2-yl)benzamide **3**, 3.5 mmol of the corresponding acyl chloride in dichloromethane (10 mL) was added. Then, 3.4 mmol N(C_2_H_5_)_3_ was added within 10 min. Within about 30 min, the reaction mixture was washed consequently with diluted HCl (1:4), Na_2_CO_3_, and H_2_O, then dried with anhydrous Na_2_SO_4_, filtered on a short column filled with neutral Al_2_O_3_, and concentrated. Spectral data confirmed the structure of diamides **4a**–**d** (Supplementary Materials, Figures S5–S20).

2-benzamido-N-(1-(3,4-dimethoxyphenyl)propan-2-yl)benzamide (**4a**): ^1^H-NMR (500 MHz, CDCl_3_) δ 1.29(d, 3H, *J* = 6.8, CH_3_), 2.88 (d, 2H, *J* = 6.4, CH_2_), 3.83 (s, 3H, OCH_3_), 3.84 (s, 3H, OCH_3_), 4.48 (dd, 1H, *J* = 8.1, 6.6, CH), 6.27 (d, 1H, *J* = 6.8, NH), 6.75–6.82 (m, 3H, Ar), 7.05 (td, 1H, *J* = 7.6, 1.5, Ar), 7.38 (dd, 1H, *J* = 7.8, 1.5, Ar), 7.48–7.52 (m, 2H, Ar), 7.53–7.55 (m, 2H, Ar), 8.04–8.05 (m, 2H, Ar), 8.78–8.80 (m, 1H, Ar), 12.07 (s, 1H, CONH); ^13^C-NMR (126 MHz, CDCl_3_) δ 168.55, 165.59, 148.95, 147.86, 139.80, 134.81, 133.58, 132.63, 131.87, 130.16, 129.98, 128.80, 127.41, 126.43, 122.85, 121.63, 121.53, 120.85, 112.58, 111.20, 55.88, 55.85, 46.62, 41.80, 20.04; FT-IR, cm^−1^: 3338 ν(-N-H, >NH-amide), 3056,3006 ν(Csp^2^-H, -Ph), 2991 ν_as_(Csp^3^-H, -OCH_3_), 2965 ν_as_(Csp3-H, -CH3), 2930 ν_as_(Csp^3^-H, -NH-CH_2_-), 2870 ν_s_(Csp^3^-H, –CH_3_), 2831 ν_s_(Csp^3^-H, –OCH_3_), ν_s_(Csp^3^-H, -CH_2_), 1679 ν(>C=O), 1627 ν(>C=O), secondary amide I, 1614 (shoulder) ν(C-C=C, -Ph_ortho_), 1592 ν(C-C=C, -Ph_1,2,4_), ν(C-C=C, -Ph_mono_), 1519 δ(N-H) and ν(C-N), trans-secondary amide II, 1519 ν(C-C=C, -Ph_mono_), 1465 (shoulder) ν(C-C=C, -Ph_ortho_), ν(C-C=C, -Ph_1,2,4_), δ_as_(-CH_3_), δ_as_(-OCH_3_), 1450 δ_s_(-OCH_3_), 1417 ν(C-C=C, -Ph_1,2,4_), 1358 δ_s_ (-CH_3_), 1296 ν(C-C=C, -Ph_ortho_), ν(C-N), secondary amide III, 1281 δ(-Csp^2^-H, -Ph_ortho_), ν(C-C=C, -Ph_1,2,4_), 1261 δ(-Csp^2^-H, -Ph_1,2,4_), 1159 δ(-Csp^2^-H, -Ph_ortho_), ρ(-CH-)/ν(N-C) in –NH-CH-, 1137 δ(-Csp^2^-H, -Ph_1,2,4_), 1091 δ(-Csp^2^-H, -Ph_1,2,4_), 1024 δ(-Csp^2^-H, -Ph_ortho_), 932 γ(Csp^2^-H, -Ph_ortho_), δ(-Csp^2^-H, -Ph_1,2,4_), 872 γ(Csp^2^-H, -Ph_ortho_-NH-), γ(-Csp^2^-H, -Ph_1,2,4_), γ(C-C=C, -Ph_mono_), 821 γ(-Csp^2^-H, -Ph_1,2,4_), 806 γ(ω)NH, 758 γ(Csp^2^-H, -Ph_ortho_-NH-); HRMS electrospray ionization (ESI) *m*/*z* calcd for [M + H]^+^ C_25_H_27_O_4_N_2_^+^ = 419.19653, found 419.19563 (mass error ∆m = −2.16 ppm).

2-chloro-N-(2-((1-(3,4-dimethoxyphenyl)propan-2-yl)carbamoyl)phenyl)benzamide (**4b**): ^1^H-NMR (500 MHz, CDCl_3_) δ 1.24 (d, 3H, *J* = 6.8, CH_3_), 2.84 (dd, 2H, *J* = 6.4, 2.9, CH_2_), 3.83 (s, 3H, OCH_3_), 3.87 (s, 3H, OCH_3_), 4.39–4.42 (m, 1H, CH), 6.16 (d, 1H, *J* = 8.3, NH), 6.73–6.76 (m, 2H, Ar), 6.82 (d, 1H, *J* = 7.8, Ar), 7.09–7.12 (m, 1H, Ar), 7.35–7.37 (m, 2H, Ar), 7.46–7.50 (m, 2H, Ar), 7.66–7.67 (m, 1H, Ar), 8.01 (dd, 1H, *J* = 7.6, 1.2, Ar), 8.76 (d, 1H, *J* = 8.3, Ar), 11.47 (s, 1H, CONH); ^13^C-NMR (126 MHz, CDCl_3_) δ 168.14, 165.50, 148.95, 147.86, 139.0 136.09, 133.42, 132.60, 132.38, 131.44, 131.37, 130.59, 129.92, 129.38, 128.68, 127.15, 126.69, 126.39, 123.44, 121.95, 121.55, 121.49, 112.55, 111.22, 55.91, 55.84, 46.56, 41.70, 19.96; FT-IR, cm^−1^: 3347 ν(-N-H, >NH-), 3064 ν(Csp^2^-H, -Ph), 2968 ν_as_(Csp^3^-H, -CH_3_), 2933 ν_as_(Csp^3^-H, -NH-CH_2_-), 2834 ν_s_(Csp^3^-H, -OCH_3_), ν_s_(Csp^3^-H, -CH_2_), 1689 ν(>C=O), 1642 ν(>C=O), secondary amide I, 1592 ν(C-C=C, -Ph_1,2,4_), 1516 δ(N-H) and ν(C-N), trans-secondary amide II, 1441 ν(C-C=C, -Ph_ortho_), δ_s_(-OCH_3_), 1416 (shoulder) ν(C-C=C, -Ph_1,2,4_), 1354 δ_s_(-CH_3_), 1316 ν(C-N), secondary amide III, 1265 δ(-Csp^2^-H, -Ph_1,2,4_), 1157 δ(-Csp^2^-H, -Ph_ortho_), ρ(-CH_2_-)/ν(N-C) in –NH-CH-, 1141 δ(-Csp^2^-H, -Ph_1,2,4_), 1050 ν(-Ph_ortho_-Cl), 1028 δ(-Csp^2^-H, -Ph_ortho_), 793 γ(ω)NH, 746 γ(Csp^2^-H, -Ph_ortho_-NH-); HRMS electrospray ionization (ESI) *m*/*z* calcd for [M + H]^+^ C_25_H_26_O_4_N_2_Cl^+^ = 453.15756, found 453.15689 (mass error ∆m = −1.48 ppm).

N-(1-(3,4-dimethoxyphenyl)propan-2-yl)-2-(4-nitrobenzamido)benzamide (**4c**): ^1^H-NMR (500 MHz, CDCl_3_) δ 1.21 (d, 3H, *J* = 6.4, CH_3_)), 2.74–2.83 (m, 2H, CH_2_), 3.74 (s, 3H, OCH_3_), 3.75 (s, 3H, OCH_3_), 4.4 (dd, 1H, *J* = 8.1, 6.6, CH), 6.09 (d, 1H, *J* = 6.8, NH), 6.64–6.74 (m, 3H, Ar), 7.05 (t, 1H, *J* = 7.6, Ar), 7.33 (dd, 1H, *J* = 8.1, 1.2, Ar), 7.47 (t, 1H, *J* = 7.8, Ar), 8.11 (d, 2H, *J* = 8.3, Ar), 8.28 (d, 2H, *J* = 8.3, Ar), 8.71 (d, 1H, *J* = 8.3, Ar), 12.41 (s, 1H, CONH); 13C-NMR (126 MHz, CDCl_3_) δ 168.39, 163.23, 149.74, 148.96, 139.63, 132.97, 129.77, 128.58, 127.35, 126.31, 123.99, 123.48, 121.55, 120.35, 112.58, 111.22, 55.87, 46.72, 41.86, 20.09; FT-IR, cm^−1^: 3329 ν_s_(-N-H, -NH-), ν(-N-H, >NH-amide), 3117, 3067, 3005 ν(Csp^2^-H, -Ph), 2970 ν_as_(Csp^3^-H, -CH_3_), 2929 ν_as_(Csp^3^-H, -NH-CH_2_-), 2868 ν_s_(Csp^3^-H, -CH_3_), 2836 ν_s_(Csp^3^-H, -OCH_3_), v_s_(Csp^3^-H, -CH_2_), 1688 ν(>C=O), 1626 ν(>C=O), secondary amide I, 1591 ν(C-C=C, -Ph_ortho_), ν(C-C=C, -Ph_para_), 1519 δ(N-H) and ν(C-N), trans-secondary amide II, ν_as_(NO_2_), 1449 ν(C-C=C, -Ph_ortho_), δ_s_(-OCH_3_), 1420 ν(C-C=C, -Ph_1,2,4_), ν(C-C=C, -Ph_para_), 1345 δ_s_(-CH_3_), 1326 ν(C-C=C, -Ph_para_), 1306, 1297 ν(C-C=C, -Ph_ortho_), ν(C-C=C, -Ph_1,2,4_), ν(C-N), secondary amide III, ν_s_(NO_2_), 1264 δ(-Csp^2^-H, -Ph_1,2,4_), 1162 δ(-Csp^2^-H, -Ph_ortho_), ρ(-CH_2_-)/ν(N-C) in –NH-CH-, 1142 δ(-Csp^2^-H, -Ph_1,2,4_), 1117 ν(NO_2_), 1031 δ(-Csp^2^-H, -Ph_ortho_), 953 γ(Csp^2^-H, -Ph_para_), 871 γ(Csp^2^-H, -Ph_ortho_), γ(-Csp^2^-H, -Ph_1,2,4_), 853 δ(NO_2_), 819 γ(Csp^2^-H, -Ph_para_), 807 γ(ω)NH, 757 γ(Csp^2^-H, -Ph_ortho_-NH-); HRMS electrospray ionization (ESI) *m*/*z* calcd for [M + H]^+^ C_25_H_26_O_6_N_3_^+^ = 464.18161, found 464.18073 (mass error ∆m = −1.90 ppm).

N-(1-(3,4-dimethoxyphenyl)propan-2-yl)-2-(2-phenylacetamido)benzamide (**4d**): ^1^H-NMR (500 MHz, CDCl_3_) δ 1.23 (d, 2H, *J* = 6.8, CH_3_)), 2.74–2.86 (m, 2H, CH_2_), 3.74 (s, 2H, CH2-Ph), 3.84 (s, 3H, OCH_3_), 3.89 (s, 3H, OCH_3_), 4.33–4.36 (t, 1H, CH), 6.02 (d, 1H, *J* = 3.9, NH), 6.71 (d, 1H, *J* = 1.5, Ar), 6.74–6.76 (m, 1H, Ar), 6.83–6.84 (m, 1H, Ar), 6.99–7.02 (m, 1H, Ar), 7.27–7.30 (m, 2H, Ar), 7.35–7.36 (m, 2H, Ar), 7.40–7.44 (m, 3H, Ar), 8.55–8.57 (m, 1H, Ar), 11.00 (s, 1H, CONH); 13C-NMR (126 MHz, CDCl_3_) δ 168.89, 168.17, 148.92, 147.85, 139.21, 134.69, 132.35, 130.04, 129.52, 129.45, 128.81, 128.74, 127.21, 126.17, 122.83, 121.56, 121.17, 112.59, 111.22, 55.92, 55.85, 46.69, 45.67, 41.67, 19.83; FT-IR, cm^−1^: 3349 ν(-N-H, >NH-amide), 3298 ν(-N-H, >NH-amide), 3063, 3030, 3008 ν(Csp^2^-H, -Ph), 2968 ν_as_(Csp^3^-H, -CH_3_), 2931 ν_as_(Csp^3^-H, -NH-CH-), ν_as_(Csp^3^-H, -CH_2_-), 2872 ν_s_(Csp^3^-H, -CH_3_), 2839 ν_s_(Csp3-H, –OCH3), ν_s_(Csp^3^-H, -CH_2_-), 1709 ν(>C=O), 1624 ν(>C=O), secondary amide I, 1587 ν(C-C=C, -Ph_1,2,4_), ν(C-C=C, -Ph_mono_), 1545, 1516 δ(N-H) and ν(C-N), trans-secondary amide II, 1516, 1496 ν(C-C=C, -Ph_ortho_), ν(C-C=C, -Ph_1,2,4_), ν(C-C=C, -Ph_mono_), 1462 ν(C-C=C, -Ph_ortho_), δ_as_(-CH_3_), δ_as_(-OCH_3_), 1446 δ_s_(-OCH_3_), 1418 ν(C-C=C, -Ph_1,2,4_), 1357 δ_s_(-CH_3_), 1296 ν(C-C=C, -Ph_ortho_), ν(C-N), secondary amide III, 1279 δ(-Csp^2^-H, -Ph_1,2,4_), δ(-Csp^2^-H, -Ph_ortho_), 1264 δ(-Csp^2^-H, -Ph_1,2,4_), 1161 δ(-Csp^2^-H, -Ph_ortho_), ρ(-CH-)/ν(N-C) in –NH-CH-, 1137 δ(-Csp^2^-H, -Ph_1,2,4_), 1100 δ(-Csp^2^-H, -Ph_1,2,4_), 1034, 1021 δ(-Csp^2^-H, -Ph_ortho_), 945 γ(Csp^2^-H, -Ph_ortho_), γ(-Csp^2^-H, -Ph_1,2,4_), 881 γ(Csp^2^-H, -Ph_ortho_), γ(-Csp^2^-H, -Ph_1,2,4_), 807 γ(ω)NH, 758 γ(Csp^2^-H, -Ph_ortho_-NH-); HRMS electrospray ionization (ESI) *m*/*z* calcd for [M + H]+ C_26_H_29_O_4_N_2_^+^ = 433.21218, found 433.21112 (mass error ∆m = −2.46 ppm).

### 2.2. In Silico Predictions

#### 2.2.1. PASS Online Predictions

The biological activity of the compounds was screened using the free web tool Prediction of Activity Spectra for Substances (PASS) Online (https://www.way2drug.com/PASSOnline/predict.php, accessed on 22 September 2024). The structural formula of the synthesized organic compounds is used by the algorithm to predict a number of possible biological activities [30,31,32].

#### 2.2.2. Theoretical Prediction of Pharmacokinetic Parameters (ADME)

Using free SwissADME online tools (http://swissadme.ch/index.php, accessed on 22 September 2024), the physicochemical characteristics, drug similarity, and pharmacokinetic parameters (absorption, distribution, metabolism, and elimination) of the produced prodrugs were examined [33].

#### 2.2.3. Theoretical Prediction of Toxicity

The ProTox-II free online program (https://tox.charite.de/protox3/, accessed on 22 September 2024) was utilized to forecast the compounds’ acute and organ toxicity. Moreover, LD50 and toxicity class estimates were obtained [34,35].

#### 2.2.4. DFT Calculations

The Gaussian 09 software package [36] was implemented in performing full geometry optimization of the molecules under study at the B3LYP/6-311G(d,p) level of theory which was found suitable in our previous work [37,38]. Overall, the lack of negative values in the subsequent frequency analysis indicated a local minimum of the potential energy surface for all the studied geometries. As a first step, a partial conformational analysis regarding the rotation around the C4-C11 (position of the CO-NH group), C15-C21 (position of the methyl group), and C21-C24 (position of the catecholate moiety) bonds for the simplest representative of the series was conducted, providing the most stable conformer for the *S*-isomer. Figure S21 illustrates the studied geometries as well as the obtained energy differences between them in terms of kcal mol^−1^. The most stable conformer was henceforth calculated for the *R*-containing enantiomer, as well as for each compound of the series, and thus considered in the following theoretical analysis. The DFT calculations provided the highest occupied/lowest unoccupied molecular orbital (HOMO-LUMO) differences necessary for retrieving quantum-chemical components such as chemical potential, chemical hardness and softness, electronegativity, and electrophilicity index according to Ref. [39]. These structures were further implemented in calculating the interaction between the compounds and albumin, interleukin-β, and muscarinic receptors M2 and M3 through molecular docking.

#### 2.2.5. Molecular Docking

A widely used approach to investigating the interaction between compounds of therapeutic value and protein molecules providing reliable results is the employment of the AutoDock 4.2.6 software package [40] in conjunction with AutoDockTools 1.5.7 [41] applied in the current research. PDB provided structures for human serum albumin, HSA, PDB entry 1AO6, interleukin-β, PDB entry 8RYS, muscarinic receptors M2 and M3, and PDB entries 4MQS and 8EA0, respectively, which were taken into consideration as macromolecules and the DFT optimized structures of the series of *S*-enantiomers (main text) and *R*-isomers (Supplementary Materials) as ligands. In all compound–protein interactions regarding the docking technique, the 3D grid size of 50 × 50 × 50 Å with a specification of 0.5 was selected. In the case of HSA, the two ligand-binding active sites named Sudlow 1 and Sudlow 2 reported in Ref. [42] were positioned in the center of the grid box, while regarding interleukin-β and the muscarinic receptors, a preliminary step of “blind docking” was performed to point out the most probable amino acid residues to interact with the ligands, followed by the conventional docking at these specific sites. The obtained results in terms of ΔG, kcal mol^−1^, were given for both enantiomers.

### 2.3. Smooth Muscle Activity

Tested animals: The study was carried out using 12-week-old male Wistar albino rats (body weight 250–280 g) obtained from the Medical University of Plovdiv, Animal Laboratory, Bulgaria. The experimental procedures were conducted with the highest confidence adhering to the Guidelines for the Care and Use of Laboratory Animals, Medical University of Plovdiv, Bulgaria, permit No. 229/09.04.2019. The procedures were approved by the current European regulations (86/609/EEC), comply with the EU Directive 2010/63/EU, and are in strict accordance with the current Institutional Animal Care Bulgaria.

Chemicals and reagents: Acetylcholine (ACh) and chemicals in Krebs solution were purchased from Sigma Aldrich (St. Louis, MO, USA). ACh and all reagents were dissolved in distilled water.

Isolated organ bath experiments: Animals were sacrificed by decapitation with anesthesia. The stomach cavity of the animals was rapidly opened. Then, the corpus smooth muscle tissues were removed delicately and placed in a Petri dish with 3–4 °C preaerated (95% O_2_–5% CO_2_) preparation solution containing NaCl/KCl/CaCl_2_ in a ratio of 27.2/1.1/1 [43]. Three circular segments (10–12 mm length, 1.1–1.3 mm width) of muscle tissue were taken from each animal, with the mucosa remaining intact. The corpus samples, as indicated by n, were equilibrated in a typical physiological salt Krebs solution containing (in mmol/L): NaCl, 140.0; KCl, 5.0; MgCl_2_, 1.2; CaCl_2_, 1.8; NaHCO_3_, 23.8; and glucose, 11.1 at a resting tension of 10 mN (1 g). Deionized water with a conductivity of 18.2 mΩ/cm^2^ was used in all experiments. Before each experiment, the pH of the buffer was determined using a HI5521 pH-meter (Hanna Instruments, Smithfield, RI, USA). The temperature of the baths was adjusted to 37 °C (±0.2 °C) by employing a thermocirculator. The tissue baths were continuously aerated with a gas mixture of 95% O_2_–5% CO_2_. The Krebs solution, containing the tissues, was refreshed every 10 min during the resting period. After the equilibration period, the active agents were precisely administered into the baths using an adjustable automatic pipette. SMPs were hung immediately on the isometric force transducer of the four-chamber tissue bath (Tissue Organ Bath System 159920 Radnoti, Dublin, Ireland) via surgical thread to measure the isometric contraction–relaxation responses. Eight SMPs were used for each group. To investigate the effect of new molecule-dependent relaxation factors, all 5 new molecules, mebeverine, and diclofenac were added to the Krebs solution with a single dose (5 × 10^−5^ M), then ACh was administered cumulatively (10^−6^ M).

### 2.4. Anti-Inflammatory Activity

#### 2.4.1. Inhibition of Albumin Denaturation

The antidenaturation assay was carried out in accordance with Milusheva et al.’s method [37,44,45]. The reaction mixture contained 0.2 mL of the tested drug dissolved in DMSO at several concentrations (20–500 μg/mL) and 0.5 mL of a 5% aqueous solution of human albumin (Albunorm 20, Octapharma (IP) SPRL, 1070 Anderlecht, Belgium). For fifteen minutes, the samples were incubated at 37 °C. Subsequently, each tube was filled with 2.5 mL of phosphate-buffered saline (pH 6.3), and the samples were heated at 80 °C for 30 min before being cooled for 5 min. Turbidity was measured at 660 nm spectrophotometrically (Cary 60 UV-Vis, Agilent Technologies, Santa Clara, CA, USA). For the blank sample, a mixture of 2.5 mL buffer and 0.2 mL DMSO was used instead of the compounds, while the product control test lacked the compounds, having 0.5 mL serum albumin and 2.5 mL buffer only. The percentage of inhibition of protein denaturation was calculated as follows:

Percentage of inhibition denaturation = [(Absorbance_control_ − Absorbance_sample_)/Absorbance_control_] × 100

The control represents 100% protein denaturation. Commercially available anti-inflammatory drugs diclofenac and acetylsalicylic acid (ASA) were used for comparison. Their anti-inflammatory effect was determined as a percentage of inhibition of albumin denaturation, following the same protocol as for the novel compounds.

#### 2.4.2. Histology and Immunohistochemistry

The histology and immunohistochemistry experiments were carried out as previously described [37].

The morphometric analysis involved tissue slices 5 μm in thickness, obtained from the circular and longitudinal layer of SM cells, as well as in the myenteric plexus of the stomach. The intensity of the immune reaction in the stomach was measured in arbitrary units (AU) on the slices immunostained for IL-1β and nNOS. The measurements were performed using the DP–Soft ver. 3.2 software, Olympus, Japan. The average intensity of pixels was recorded in arbitrary units in the range of 0–256 on microphotographs of the stomach, 0 being black and 256 being white. A minimum of 50 points were measured in the stomach at magnification ×400. All measurements involved five slices per animal and an examination of all cross-sections of the stomach.

### 2.5. Statistical Analysis

The SPSS 23.0 program (SPSS Inc., Chicago, IL, USA) was used for statistical analysis. All data are expressed as mean ± standard deviation (SD). The number of tissue preparations used in each experiment is indicated by n. Statistical significance between two independent groups was analyzed by independent sample *t*-test. The statistical significance level was considered as *p* < 0.05.

## 3. Results and Discussion

### 3.1. Synthesis of Mebeverine Derivatives

**Figure 1:**
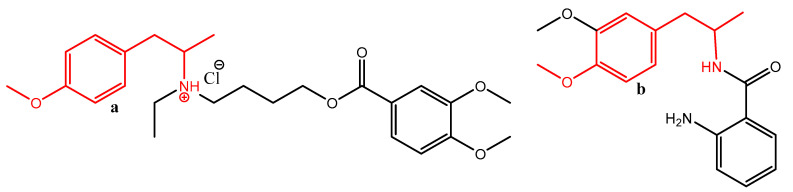
Structure of (**a**) mebeverine hydrochloride; (**b**) mebeverine derivative. Common structure in red.

The current work aims to synthesize novel more efficient mebeverine derivatives for IBS treatment and to compare their biological activities with NSAID diclofenac and a muscle relaxant mebeverine (Figure 1). Predicted by software, spasmolytic and anti-inflammatory activities were validated using in vitro or ex vivo experiments. This makes it feasible to research the pharmacological activity and gather information on the duration of the pharmacological effects and the mechanisms of activity.

**Scheme 1:**
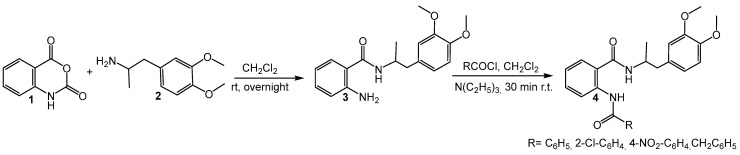
Synthesis of mebeverine derivatives **3**, **4a**–**d**.

Our synthetic methodology was based on the previously described reaction [37,46,47,48] of isatoic anhydride with 2-phenylethylamines. Herein, we describe our ongoing investigation into the reaction of isatoic anhydride **1** with previously reported 1-(3,4-dimethoxyphenyl)propan-2-amine **2** [28]. The product, 2-amino-N-(1-(3,4-dimethoxyphenyl)propan-2-yl)benzamide **3**, was obtained with 90% yield, according to Scheme 1. The structure of the resultant molecule was confirmed by FT-IR, ^1^H-NMR, ^13^C-NMR, and MS spectra and all the signals were completely consistent with the corresponding molecule (Supplementary Materials, Figures S1–S4). The obtained benzamide **3** was applied in the synthesis of amides. The amide group exists in biological systems and has a crucial role in medicinal chemistry [49,50,51]. Using acylation, we obtained diamides **4a**–**d** (Table 2).

The structures of all the compounds were validated by spectral data (Supplementary Materials, Figures S1–S12) and were completely consistent with the corresponding molecules.

### 3.2. In Silico Calculations

#### 3.2.1. ADME Profile of the Studied Compounds

Using in silico methods, the drug-likeness of the compounds and their ADMET properties have also been predicted theoretically. Increased lipophilicity, membrane permeability, and pharmacological activity are the outcomes of conformational restriction of small drug molecules brought about by the incorporation of this non-covalent interaction into drug design [30].

According to Lipinski’s rule of five [52], the ADME investigation demonstrates that the compounds have good GI absorption except **4c**. We found that **3** met the standard values (Table 3) of molecular weight (MW < 400), XLOGP3 ≤ 5.2, ESOL or estimated solubility (log S: not more than 6), polarity (TPSA: 20 to 130 Å^2^), flexibility (RB: no more than 9), etc. The amides **4a**–**d** met most criteria except (Table 3) MW > 350, rotors > 7, and XLOGP3 > 3.5. The bioavailability score for all the substances is 0.55, indicating good oral absorption [52]. Additionally, they score well on the synthetic accessibility scale (2.61–3.50), a crucial factor in drug discovery. All the compounds exhibit good skin permeability, with log Kp ranging from −5.80 to −5.12.

Cytochrome P450 (CYP) inhibition results in pharmacological toxicity [53,54]. The examined substances are not anticipated to function as P-glycoprotein substrates. According to the calculations, every amide **4** should inhibit the CYP2C19, CYP2C9, CYP2D6, CYP3A4 (marked with “+” sign in the Table 4), with the exception of the CYP1A2 isoform (marked with “–” sign in the Table 4); while compound **3** is predicted to inhibit all isoforms (Table 4).

According to ProTox-II acute toxicity data, all of the substances have a toxicity class 4 and compound **3** only is in class 5 (on a 1–6 scale, where 1 is highest and 6 is lowest toxicity). The estimated LD_50_ for compounds **4a**, **4c**, and **4d** is 2000 mg/kg, compound **3** only has LD_50_ = 2500 mg/kg, and **4b** LD_50_ = 1000 mg/kg.

Based on the calculated results, each compound can be assumed as a potential therapeutic candidate.

After calculating the low toxicity level of the synthesized 2-amino-N-(1-(3,4-dimethoxyphenyl)propan-2-yl)benzamide **3** and its amides **4**, we focused on investigating their spasmolytic and anti-inflammatory activities, predicted by software tool PASS Online.

#### 3.2.2. Density Functional Theory (DFT) Calculations

**Figure 2:**
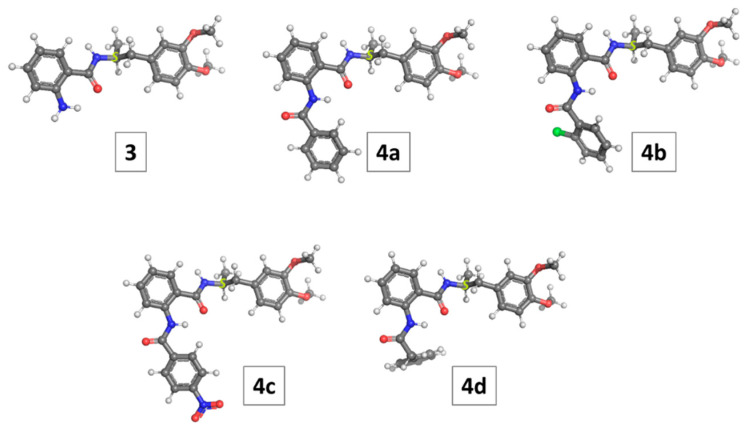
B3LYP/6-311G(d,p) optimized structures of the (*S*)-enantiomers of the series of compounds under study.

The studied compounds were subjected to density functional theory (DFT) optimization in order to theoretically analyze their properties. The optimized structures of the (*S*)-enantiomers are presented in the following Figure 2, whereas the corresponding (*R*)-enantiomers are illustrated in Figure S22.

**Figure 3:**
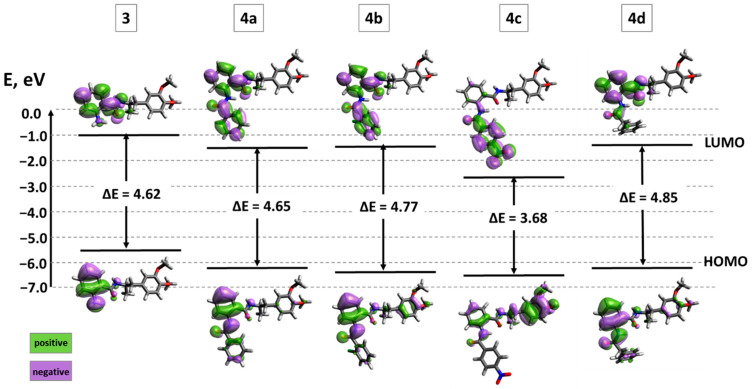
The highest occupied and lowest unoccupied molecular orbitals (HOMO/LUMO) for the (*S*)-enantiomers of the compounds from the series.

Further implementation of the DFT calculations provides information about the highest occupied/lowest unoccupied molecular orbital (HOMO-LUMO) values, their distribution on the moieties in each molecule, and the dependence on those quantum-chemical parameters [55] presented for the (*S*)-enantiomers in Figure 3 and Table 4, respectively, and in Figure S21 and Table S1 for the (*R*)-enantiomers, correspondingly. The obtained data show that the electron cloud of HOMO is mainly located on the benzamide moiety in compounds **3** and **4a**; on the catecholate part in compounds **4b** and **4c**; and the entire molecule in compound **4d**. The electron cloud of LUMO, on the other hand, is mainly situated on the benzamide moiety in all studied structures. Nevertheless, a parameter of immense significance is the HOMO-LUMO energy gap revealing the chemical stability and reactivity of the series. The larger the gap, the greater the reactivity but the lower the stability, so the studied compounds are ranked **4c** (3.66) > **3** (4.62) > **4a** (4.65) > **4b** (4.78) > **4d** (4.85) concerning their stability, but in a reversed order regarding the expected chemical reactivity.

The calculated HOMO and LUMO energy values give further evidence of the electronegativity, chemical potential, chemical hardness and softness, and the electrophilicity index, presented in the following Table 5. Overall, the negative values of the chemical potential Pi of all compounds under study indicate their stability: they are not expected to spontaneously break down to their original elements [56]. Furthermore, the biological activity strongly depends on the softness/hardness of a molecule, e.g., soft compounds are expected to interact more easily with proteins as suggested by the hard–soft–acid–base (HSAB) approximation [57]. Hence, the calculated values for the chemical softness σ for **3**, **4a**, **4c**, and **4d** staying close and in the range of 0.41–0.43 eV suggests that these compounds should be able to interact much more easily with molecules of biological origin as compared to the Cl-containing **4c** which has a σ of 0.55 eV, firmly deviating from the series. The high electrophilicity index of **4c**, on the other hand, outlines it as the best electron density acceptor in the series with estimated ω of 5.83 eV, whereas the rest of the compounds in the series appear to be prone to supplying electrons as evidenced by their lower ω values weakening in the order **3** < **4d** < **4b** < **4a** (2.37 eV < 3.02 eV < 3.21 eV < 3.23 eV).

#### 3.2.3. Molecular Docking Simulation

**Figure 4:**
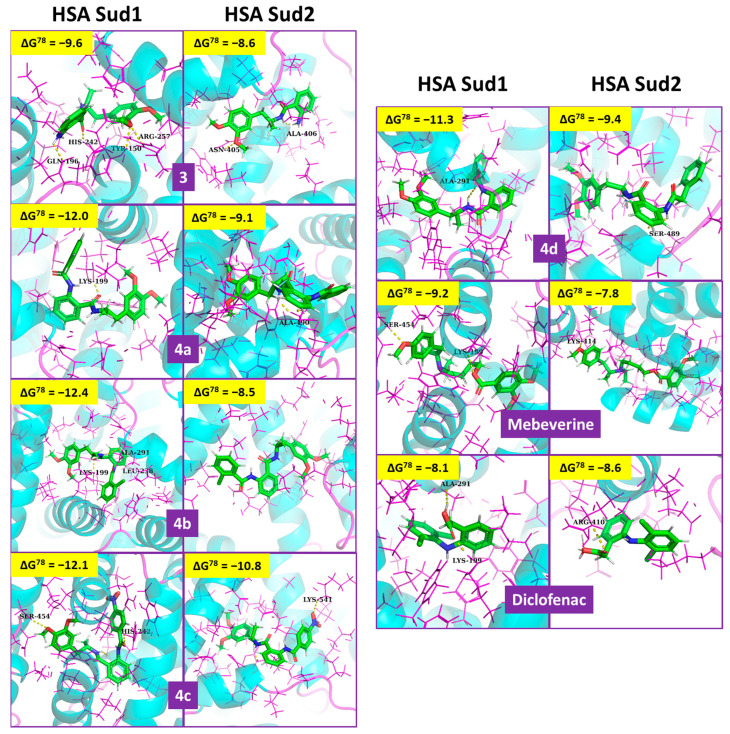
The two ligand-binding centers of human serum albumin (HSA) known as Sudlow 1 and Sudlow 2, (HSA Sud1 and HSA Sud2), and the corresponding lowest estimated Gibbs energies of interaction in kcal mol^−1^ with the (*S*)-enantiomers of the compounds from the series 3 to 4d, as well as mebeverine and diclofenac obtained through molecular docking via AutoDock 4.2. The polar interactions between the ligands and the residues from the surroundings are presented by the indication of the three-letter code for the amino acid/acids.

The final step in the preliminary in silico predictions is the implementation of molecular docking for analyzing the interaction between the ligands from the series and the protein molecules of albumin (two known active sites), the muscarinic receptors MR2 and MR3, as well as interleukin-β (IL-β). The obtained results in terms of Gibbs energies of interaction in kcal mol^−1^ are presented in Figure 4 and Table 6 for the (*S*)-enantiomers and in Figure S23 for (R)-enantiomers of the compounds.

The obtained results indicate that all studied compounds should exert a well-pronounced anti-inflammatory effect due to their strong binding to albumin as all calculated Gibbs energies stay firmly on negative ground. Noteworthily, the active site denoted as Sudlow 1 is more prone to binding the molecules as compared to Sudlow 2, evidenced by the greater absolute value change of ΔG (compare data in Figure 4). Although the results stay close, the amides **4a** to **4d** appear slightly better suited to interacting with the protein in comparison to ligand **3** (ΔG ranging from −12.4 to −11.3 kcal mol^−1^ as opposed to −9.6 kcal mol^−1^), which could be attributed to the functionalization of the amino group, especially with the electronegative Cl, and NO_2_ leading to greater interaction with the surrounding amino acid residues. Noteworthily, the DFT-obtained parameter “softness” (Table 5) suggests that among all mebeverine derivatives, the nitro-containing structure should interact to the greatest extent with biological molecules, being the “softest”, according to the hard–soft–acid–base (HSAB) approximation. Additionally, calculations with the molecules of diclofenac and mebeverine were performed. The computations further imply that the newly synthesized mebeverine derivatives are expected to exert a more pronounced anti-inflammatory effect in comparison to mebeverine and diclofenac, indicated by the greater absolute value change in Gibbs energies for binding both centers of albumin (compare data presented in Figure 4). Therefore, the resulting more stabilized albumin–ligand complex is less susceptible to denaturation. Moreover, the two muscarinic receptors and interleukin-β are expected to successfully bind the molecules under study, evidenced by the negative change in the calculated Gibbs energies. Among the two muscarinic receptors, MR2 appears more prone to binding the compounds of the series (indicated by the about 2 kcal mol^−1^ higher absolute value ΔG), in addition to results for MR3 that do not indicate a preferred interaction between the ligands and the receptor as the obtained changes in Gibbs energy stay close, between −9.3 and −10.7 kcal mol^−1^, falling within the limits of the method. Analogous conclusions could be drawn for the interaction between the studied compounds and interleukin-β being in the range of −10.5 and −12.2 kcal mol^−1^. Overall, the molecular docking calculations undoubtedly imply the positive interaction between the compounds from the series and the studied specific receptors/cytokine, hence providing initial theoretical evidence of their expected therapeutic effect.

### 3.3. Biological Evaluation of 2-amino-N-(1-(3,4-dimethoxyphenyl)propan-2-yl)benzamides

#### 3.3.1. Spasmolytic Activity

Activating or inhibitory effects of the target compounds on muscle activity in isolated smooth muscle (SM) models from the stomach or intestinal wall are suitable ex vivo models to study pharmacological concentration–response correlations. According to Russell and Birch’s method [58], these ex vivo experiments can be a valuable instrument in the fundamental study of several novel drugs. This helps to minimize the use of animals in investigations. The tissue bath models account for a change in the electrical, pharmacological, or electropharmacological response of treated SM segments, exposed to agonists and antagonists alone or in combination.

**Figure 5:**
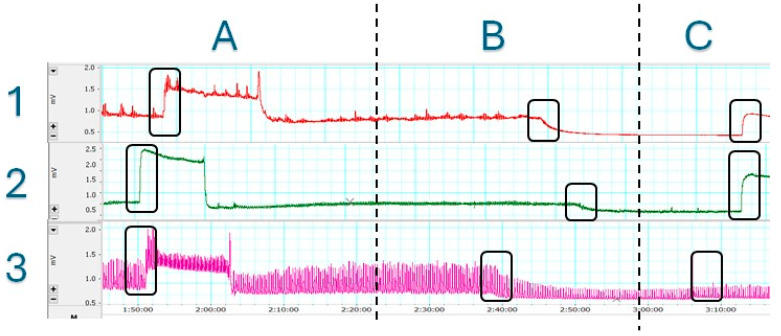
Typical experimental record, showing changes in SM contractile activity after applying 1: **4b**, 2: Diclofenac, and 3: Mebeverine. Zone A represents the effect caused by ACh (10⁻⁶ M); Zone B represents the effects of the tested compounds; Zone C represents the effect caused by ACh (10⁻⁶ M) in the presence of the tested compounds.

A balanced Krebs salt solution was employed as the standard in the model SM system to examine isolated muscle preparations evaluated ex vivo. By preserving osmotic pressure and offering a nutritional medium, it can meet the fundamental survival requirements of organs or cells in vitro and has the characteristics of a buffer solution (pH regulation). Concentrations of potassium, sodium, magnesium, calcium, chloride, and phosphate salts are similar to those in the extracellular fluid, which is relevant in studies involving muscle contraction because contraction/relaxation cycles are highly dependent on ion gradients. By monitoring concentration-dependent changes in isometric contraction following the test substance’s effect on Krebs-incubated SM, the strength of the effect can be determined. Therefore, the present study aimed to establish the level and nature of a pharmacologically treated model of gastric smooth muscle. The main excitatory neurotransmitter acetylcholine is known to be suitable for this purpose [59]. In our previous study, we demonstrated that acetylcholine and its analog carbachol induce stable contraction in rat gastric SMs [38]. After cholinergic stimulation, SM preparations respond with an initial spike component (phasic contraction) due to the increased level of ionized cytosolic calcium. This is followed by a persistent, sustained tonic contraction caused by extracellular calcium influx (Ca^2+^ influx) in SM cells [60]. In the current study, the synthesized mebeverine derivatives enable the investigation of dose–response interactions. The gastric smooth muscle model is appropriate for assessing the agonistic and antagonistic effects of novel derivatives that have been produced because of the several paths through which their influence may result in smooth muscle contraction or relaxation. The current investigation examined the isometric recording of changes in the spontaneous contractile activity of SM preparations for **3** and **4a**–**d**, in comparison to mebeverine and the known anti-inflammatory agent diclofenac. We found that all the compounds showed an inhibitory effect on the amplitude, tone, and frequency of the SM preparations’ contractile response when administered independently (Table 7). The frequency of spontaneous contractions showed a statistically significant increase of 26% for **4c**. The SM’s tone changed most noticeably with **4b** (Figure 5, zone B), changing by 54%, and the contraction amplitude decreased most with **3** and **4c**, decreasing by more than 66% (Table 7).

We noticed similarities of the relaxation with varying amplitude and strength of tonic reaction for all the compounds (Figure 5, zones B1–B3). As a result, the relaxation effects are entirely consistent with the antispasmodic effects predicted by the in silico methods.

After applying the synthesized mebeverine derivatives to an organ bath containing the neurotransmitter (Ach, 10^−6^ M), the biological reaction was evaluated. We discovered an intriguing fact: in SM preparations previously treated with mebeverine, we observed a partial preservation (54%) of the spontaneous phasic activity of the isolated muscle segments following exposure to the neurotransmitter and a complete absence of the tonic component (100% reduction) in the ACh reaction (Figure 5, zones A–C). Even after repeatedly washing the separated muscle tissues in Krebs buffer solution for 45 min, the effect remains intact. The idea that there is an irreversible inhibitory effect on the acetylcholinergic system is supported by these results. Mebeverine has been used in clinical practice for years for IBS treatment as a direct SM relaxant but that indicated a significant side effect of this drug. This complete absence of an acetylcholine reaction in the presence of mebeverine may be associated with its irreversible inhibition of acetylcholinesterase [61]. The action of mebeverine on the acetylcholine response may be due to its antagonistic potential on muscarinic receptors [62]. Out of the five metabotropic muscarinic receptors that are currently found in smooth muscles, M1 and M3 are the most highly expressed and are mediated by phospholipase C [63]. They can affect spontaneous contractions directly or indirectly, altering their tone and amplitude.

On the other hand, the tonic ACh response changed with **3**, **4a**–**d**, and diclofenac from 33% to 59% (Figure 5, **4b**: zones A–C; **diclofenac**: zones A–C). This rules out the possibility that their actions are only influencing cholinergic mediation throughout the ACh response, which is an extracellular rise in cytosolic Ca^2+^ concentration leading to SM contraction. Through the parasympathetic nervous system, ACh itself has a proinflammatory impact, which inhibits inflammation and helps to regulate it. In IBS, an inflammatory impact is seen as a result of the loss of ACh-mediated inflammatory inhibition [64].

Certain IBS-related diseases, particularly stress and anxiety disorders, result in a suppression of choline acetyltransferase production in the stomach and brain, which favors the synthesis of acetylcholinesterase. This process is associated with an inflammatory effect due to the loss of ACh-mediated inflammatory inhibition [61]. The action of the neurotransmitter ACh is sharp but short due to the rapid inactivation of the mediator by acetylcholinesterase [62]. In our research, we found that the anti-inflammatory drug diclofenac and all newly synthesized molecules only partially affect these processes, but do not inhibit them completely. These partial effects of diclofenac can be attributed to its function as a concurrent inhibitor of cholinesterase [65]. Therefore, the current work defines the target process for upcoming experimental approaches to influence the cholinergic-mediated system by acting on novel compounds as possible pharmaceutical products. The precise mechanisms of these processes still need to be clarified.

#### 3.3.2. Anti-Inflammatory Effect

##### In Vitro Inhibition of Albumin Denaturation

Novel molecules were evaluated in vitro and ex vivo for their anti-inflammatory effect. Anti-inflammatory medications decrease inflammation and discomfort. To treat inflammation, two types of pharmaceuticals are commonly used: non-steroidal anti-inflammatory drugs and steroids. Many negative side effects are associated with non-steroidal medications, including GI issues that may cause stomach ulcers [45,66,67].

Diclofenac and ASA, as known anti-inflammatory drugs, were compared to the novel amides assessing their ability to prevent contractions and inflammation in the GI tract.

**Figure 6:**
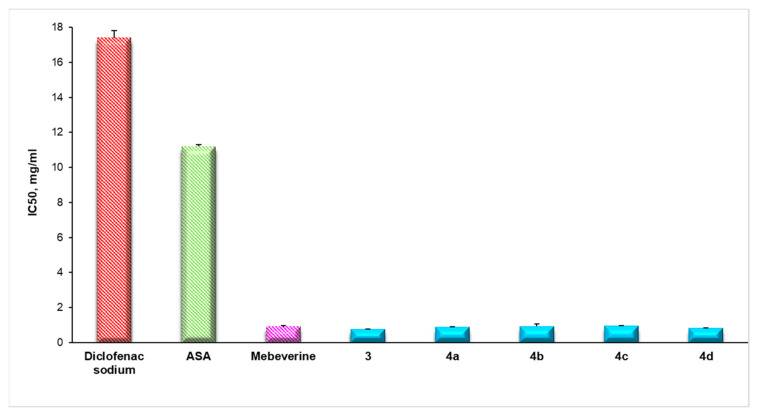
IC_50_ Inhibition of albumin denaturation (in mg/mL) of newly synthesized hybrids (blue) compared to mebeverine (purple). Diclofenac (red) and ASA (green) were used as standards.

We measured the resistance to albumin denaturation by applying the newly synthesized compounds, compared to mebeverine and anti-inflammatory drugs diclofenac and ASA (Figure 6). The newly synthesized 2-amino-N-(1-(3,4-dimethoxyphenyl)propan-2-yl)benzamine and its amides demonstrated a very good anti-inflammatory activity in preventing albumin denaturation compared to ASA and diclofenac sodium.

Amine **3** (0.76 mg/mL), as well as amides **4a** (0.87 mg/mL) and **4d** (0.83 mg/mL), had lower IC_50_ compared to mebeverine (0.91 mg/mL), and therefore better anti-inflammatory activity, while **4b** (0.93 mg/mL) and **4c** (0.94 mg/mL) had similar IC_50_ to mebeverine (Table 8). All the compounds have better activity than well-known drugs ASA and diclofenac.

Further ex vivo experiments were carried out to verify these findings and assess the impact of the hybrids on nNOS and interleukin-1 (IL-1) expression.

##### Ex Vivo Immunohistochemical Analysis

One of the signaling molecules produced during the inflammatory process and participating in it is IL-1β. The production of this cytokine is very rapidly induced by acute inflammatory reactions associated with infections, trauma, neoplasia, stress, brain death, and various types of tissue damage.

**Figure 7:**
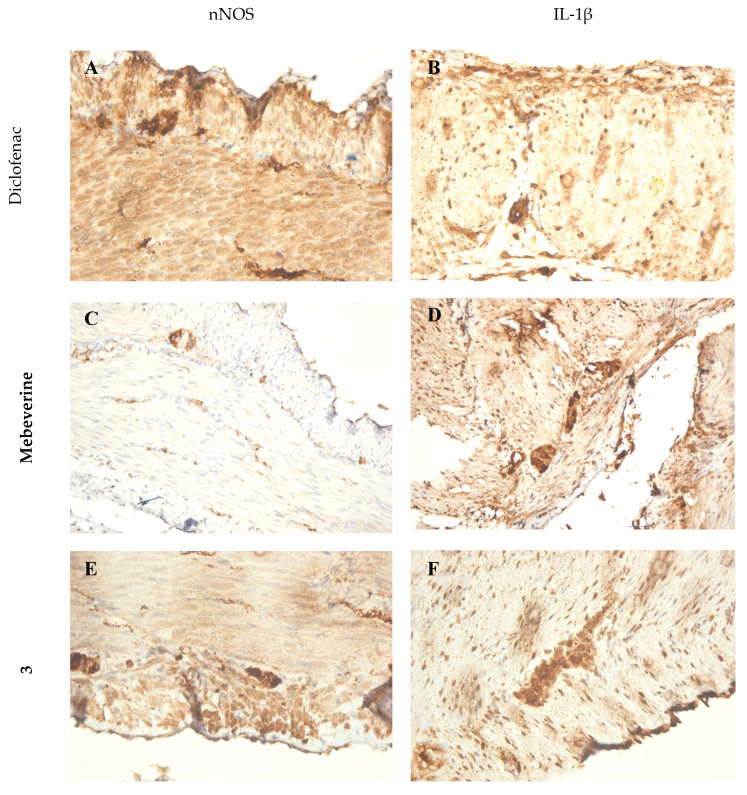

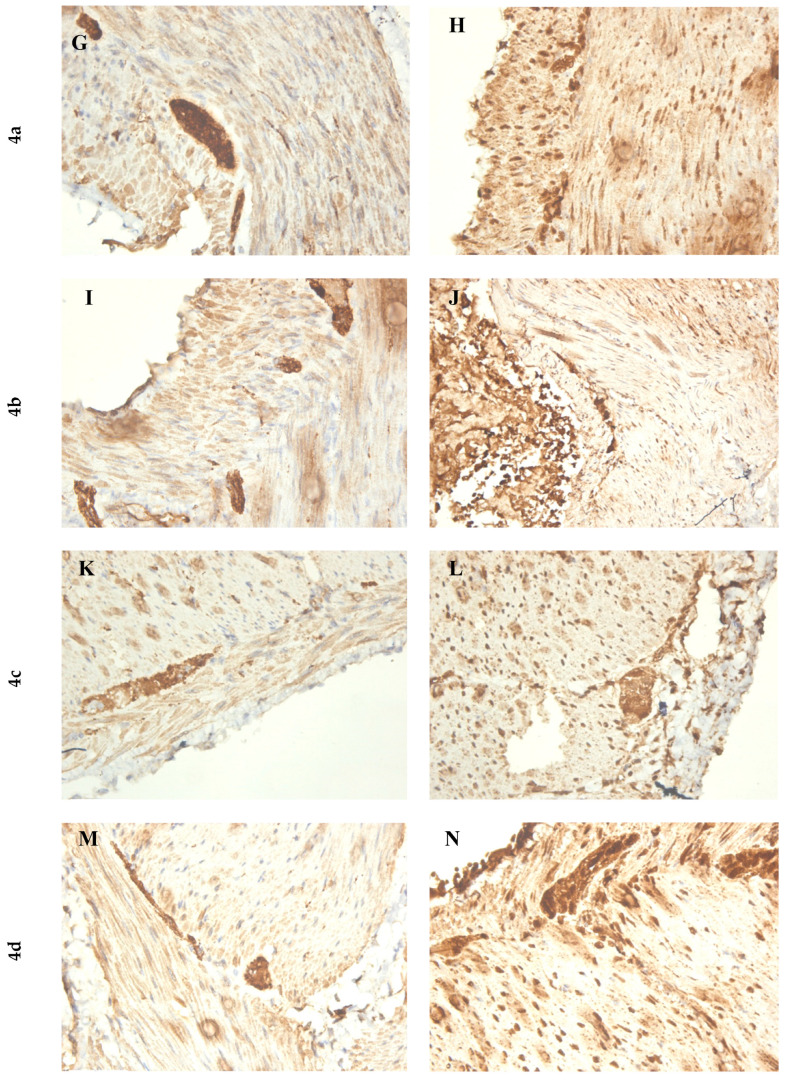
Microphotographs of SM preparations from the rat corpus incubated with diclofenac, mebeverine, and newly synthesized mebeverine derivatives **3**, **4a**–**d** for 1 h. (**A**) Diclofenac increased expression of nNOS, ×400; (**C**,**I**,**K**,**M**) Preparations incubated with mebeverine, **4b**, **4c**, and **4d** showed weak expression of nNOS, ×400; (**E**,**G**) Preparations incubated with substances **3** and **4a**, well-stained SM cells, and neurons in the myenteric plexus with nNOS, ×400. (**B**) Preparations incubated with diclofenac, well-stained for IL-1β, ×400. (**D**) Preparations incubated with mebeverine, good expression of IL-1β, ×400. (**F**,**H**,**J**) Preparations incubated with substances **3**, **4a**, and **4b**, good expression of IL-1β, ×400; (**L**) Preparations incubated with substance **4c**, weak expression of IL-1β, ×400; (**N**) Preparations incubated with substance **4d**, increased expression of IL-1β, ×400.

We found that diclofenac, mebeverine, and **3**, **4a**–**d** showed a significant change in the intensity of the immunoreaction for IL-1β and nNOS. The measured levels for IL-1β in the preparations incubated with diclofenac were 165.9 (159.7–172 AU) and those treated with mebeverine were 175.5 (165–185.9 AU, *p* < 0.05). Similar values were reported after treatment with substance **3**, 160.5 (154.7–166.3 AU), and **4a**, 164.3 (159.4–169.2 AU). The intensity of IL-1β immunoreaction was significantly reduced after treatment with substance **4b**, 150.1 (143–157.2 AU, *p* < 0.05), and **4c**, 121 (113.2–128.9 AU, *p* < 0.05). Increased intensity of the immunoreaction for IL-1β after treatment with **4d**, 177.5 (169.1–185.9), was observed (Table 9, Figure 7).

The immune response data for nNOS, on the other hand, showed a drastic difference for diclofenac, 172.2 (166.4–177.9 AU, *p* < 0.05), compared to Mebeverine, 117.6 (112.1–123 AU). We found that compound **4c**, 116.4 (112.7–120 AU), has a similar inhibition to mebeverine, followed by **4c**, 143.8 (123.2–164.5 AU, *p* < 0.05), and **4d**, 135.1 (130.4–139.9 AU, *p* < 0.05), that have statistically higher inhibition than mebeverine. Compound **4a**, 175.5 (167.8–183.2 AU, *p* < 0.05), had inhibition significantly higher than mebeverine but comparable to diclofenac.

We found that diclofenac strongly inhibited IL-1β production in SM cells and neuronal cells in gastric SM preparations, at concentrations up to 100 μM (Figure 7B). At the same time, it increases the synthesis of nNOS, possibly by stimulating the activity of the NO-sensitive guanylyl cyclase (Figure 7A).

Comparing the activity of compounds **3**, **4a**–**d** to diclofenac, we found a lower activity regarding the expression of nNOS in SM cells, but close strength and density of the reaction in neuronal cells. The expression of IL-1β in the preparations incubated with substances **3**, **4a**, and **4c** is close to that with diclofenac. However, the newly synthesized substance **4d** has a higher proinflammatory activity than diclofenac in terms of IL-1β expression in the studied SM preparations. Substances **3**, **4a**, and **4c** have IL-1β expression similar in intensity to mebeverine.

Mebeverine, on the other hand, showed a weaker activity than diclofenac, expressed in a weaker expression of nNOS, but the intensity of IL-1β expression is higher in neuronal cells from SM preparations. The expression data of the newly synthesized substances **3**, **4a**, **4b**, and **4d** showed that they induce a more intense expression of nNOS in neuronal cells compared to mebeverine.

The results of the comparative study of the novel mebeverine derivatives regarding the expression of nNOS confirmed previously reported data that NO can significantly increase the resistance of the gastrointestinal mucosa to injury [68]. Numerous studies confirmed these observations and established a role for NO in the activation of several elements of “gastric mucosal defense”, including secretion of mucus and bicarbonate, reactive hyperemia, and formation of a “mucoid plug” at sites of epithelial injury, which promotes rapid reabsorption and epithelialization after injury [69].

The anti-inflammatory activity of mebeverine we found is consistent with earlier studies by El-Haggar et al. in patients [18].

The better effect of diclofenac in reducing the expression of IL-1β compared to mebeverine is probably due to the fact diclofenac acts at the molecular level by inhibiting COX and blocking the synthesis of prostaglandins, thus in inflammation both IL-1β and COX 2 are increased [70] as major components of the inflammatory response [71].

This study demonstrates the effectiveness of newly synthesized compounds **3**, **4a**–**d** that exhibit anti-inflammatory properties similar to classic non-steroidal anti-inflammatory agents such as diclofenac, as well as similar to the known antispasmodic mebeverine.

The need to search for new molecules with anti-inflammatory properties is dictated by the large number of side effects that classic non-steroidal anti-inflammatory agents have with long-term use, which is very harmful to patients. From the study, we can conclude that all the newly synthesized substances showed a closer anti-inflammatory activity to diclofenac expressed in lowering the activity of IL-1β. On the other hand, the stimulated expression of nNOS by the newly synthesized substances is closer to the activity of mebeverine.

Further investigation of the intracellular pathways is needed to confirm these observations.

Based on the obtained results, we can conclude that novel compounds have the antispasmodic potential of mebeverine and anti-inflammatory effect of anthranilic acid residue. Future in vivo testing is needed to confirm those effects. Absorption, distribution, metabolism, and elimination of the compounds should be studied to determine their degradation and the necessary concentration in the tissues, especially in the gastrointestinal tract. Short-term and long-term toxicological analysis in animal models also is necessary. A deeper understanding of the molecular mechanism of the most active compounds **3**, **4a** and **4b**, especially their association with specific receptors and enzymes, is important to confirm their efficacy and safety. Evaluation of effects on specific receptors, enzymes, and signaling pathways, as well as investigation of long-term efficacy and possible development of tolerance with prolonged use, is needed.

## 4. Conclusions

A series of novel mebeverine analogs were obtained. Based on the in silico data, their spasmolytic and anti-inflammatory effect was tested. Compounds **3**, **4a**, and **4d** had lower IC_50_ compared to diclofenac and ASA and therefore better anti-inflammatory activity in preventing albumin denaturation.

Modulating the signaling pathways associated with SM contractility, particularly those most prominently expressed in compounds **4a**, **4b**, and **4c**, represents a promising new strategy in the management of gastrointestinal disorders. These pathways play a crucial role in controlling the contractile responses of smooth muscle, which are key contributors to conditions like irritable bowel syndrome (IBS) and other functional gastrointestinal disorders. By targeting these specific pathways, it may be possible to alleviate symptoms such as abdominal pain and discomfort more effectively, while also reducing the occurrence of adverse side effects commonly associated with current treatments, such as mebeverine.

In addition to their effects on SM contractility, compounds **4a**, **4c**, and **4d** have shown significant potential in modulating the inflammatory response within the gastrointestinal tract. The ability to influence both contractility and inflammation provides a dual-action approach, offering a comprehensive solution for the prevention and treatment of these conditions.

Among the tested compounds, **3**, **4a**, and **4b** have emerged as the most active and effective in all the studies conducted. The detailed understanding of their molecular mechanism of action, which includes effects on both smooth muscle contractility and inflammatory pathways, provides a solid foundation for the development of new therapeutic products. The findings presented here underscore the importance of further research on these three compounds, as they hold significant promise for improving the quality of life for individuals suffering from IBS and related gastrointestinal disorders. The development of novel treatments based on these mechanisms could lead to more targeted and effective therapies, with fewer side effects, thus addressing a substantial unmet need in the management of gastrointestinal health. Future in vivo and preclinical experiments will contribute to the establishment of these novel mebeverine derivatives as potential drug candidates against inflammatory diseases in the gastrointestinal tract.

## Figures and Tables

**Table 1:** Common medications for IBS treatment.

Drug Class	Mechanism of Action	Potential Adverse Effects
Bulking Agents		
Natural (derivatives of agar, psyllium, kelp, plant gums, ispaghula husk) Synthetic (methylcellulose, carboxymethylcellulose)	Long-chain carbohydrates swell in intestinal fluid; Leads to softer bulkier stools; Promotes peristalsis and eases intestinal transit	Aggravates abdominal pain or bloating, therefore is not recommended for patients with bloating and diarrhea; Hinders the absorption of concurrently administered drugs
Laxatives		
Stimulant laxatives (bisacodyl, senna, danthron, ricinoleic acid)	Stimulates motility using non-specific local irritation or a selective action on the intramural nerve plexus of intestinal SM	Severe cramping, fluid loss, malabsorption, and hypokalemia
Osmotic laxatives (magnesium salts, lactulose, sorbitol, polyethylene glycol)	Increases intraluminal water content (osmotic effect); Causes distension of the bowel and stimulates colonic peristalsis	Abdominal cramping, flatulence, diarrhea
Stool softeners (docusate)	Facilitates uptake of intestinal fluids	Abdominal cramping and diarrhea
Antidiarrheal agents		
Opioid agonists (loperamide, diphenoxylate-atropine, codeine)	Stimulates m-opiate receptors in the enteric nervous system; Inhibits peristalsis; Increases fluid absorption secondary to prolonged intestinal transit; Increases basal and sphincter pressure	Diphenoxylate-atropine and codeine cross the blood–brain barrier; Codeine may cause dependence
Tricyclic antidepressants		
Tricyclic antidepressants (TCAs) (amitriptyline, desipramine, clomipramine, doxepin, trimipramine)	Block reuptake of norepinephrine and serotonin and exhibit anticholinergic activity; Modulate sensorimotor activity and GI transit; Reduce pain	Fatigue, somnolence, dry mouth, urinary retention; Aggravates constipation; therefore it is not recommended for patients with IBS-C or IBS-A; High rate of discontinuation due to adverse effects
Selective serotonin reuptake inhibitors (fluvoxamine, luvoxamine, sertraline, paroxetine)	Selective blockade of serotonin reuptake	Better safety than TCAs; Insomnia, night sweats, weight loss, and sexual dysfunction
Benzodiazepines		
Benzodiazepines	Antianxiety effect; Mitigates exacerbation of IBS Symptoms by psychological distress	Potential for physical dependence; Interactions with other drugs; Should be used on a limited basis
Serotonergic agents		
5HT4 agonist (tegaserod)	Accelerates GI transit; Reduces visceral sensitivity and hypersensitivity; Enhances peristalsis; Increases gastric emptying and compliance; Enhances fluid secretion	Headache and mild transient diarrhea Rare case of ischemic colitis (transient and without long-term sequela) reported during postmarketing surveillance
5HT3 antagonist (alosetron)	Slows orocecal and colonic transit; Enhances intestinal absorption	Constipation in more than 20% of patients; Ischemic colitis; Contraindicated in patients with IBS-C
Antispasmodic agents		
Anticholinergic agents (dicyclomine, hyoscyamine, cimetroprium bromide, belladonna, propantheline)	Reduces the excessive contractility of SM cells	Some of these agents have not undergone rigorous scientific testing; Only dicyclomine and hyoscyamine are approved for the treatment of IBS patients
Calcium channel blockers (pinaverium bromide, otilonium bromide, mebeverine)	Reduces the excessive contractility of SM cells	Some of them are superior to placebo in relieving abdominal pain in patients with IBS; Mebeverine works by controlling bowel movements and relaxing the muscles of the intestines
Opioid agonists (trimebutine)	Reduces the excessive contractility of SM cells	Superior to placebo in relieving abdominal pain in patients with IBS

**Table 2:** Synthesis of mebeverine derivatives.

4	R	Yield, %	mp, °C
**a**	C_6_H_5_	89	152.8
**b**	2-Cl-C_6_H_4_	88	oil
**c**	4-NO_2_-C_6_H_4_	39	178.4
**d**	CH_2_-C_6_H_5_	85.6	107.2

**Table 3:** ADME profile of the compounds **3**, **4a**–**d**.

Compound	MW, g/mol	log S (ESOL)	Log P_o/w_	Log Kp, cm/s	TPSA, Å^2^	H-Acceptor	H-Donor	RB	Csp^3^	GI Absorption
**3**	314.38	−3.86	2.78	−5.80	73.58	3	2	7	0.28	High
**4a**	418.48	−5.30	4.01	−5.36	76.66	4	2	10	0.20	High
**4b**	452.93	−5.90	4.50	−5.12	76.66	4	2	10	0.20	High
**4c**	463.48	−5.37	3.45	−5.75	122.48	6	2	11	0.20	Low
**4d**	432.51	−5.27	4.18	−5.49	76.66	4	2	11	0.23	High

MW: Molecular weight, ESOL LogS: Estimated aqueous solubility, Log P_o/w_: Octanol/water partition coefficient, log Kp: Skin permeation, TPSA: Topological polar surface area, H-acceptor: Number of hydrogen bond acceptors, H-donor: Number of hydrogen bond donors, RB: Rotatable bonds, Fraction Csp^3^: Ratio of sp^3^ hybridized carbons over the total carbon count, GI absorption: Gastrointestinal absorption.

**Table 4:** Inhibition of CYP isoforms.

Compound	CYP1A2	CYP2C9	CYP2C19	CYP2D6	CYP3A4
**3**	+	+	+	+	+
**4a**	–	+	+	+	+
**4b**	–	+	+	+	+
**4c**	–	+	+	+	+
**4d**	–	+	+	+	+

**Table 5:** Quantum-chemical descriptors obtained through DFT calculation of the HOMO/LUMO values at the B3LYP/6-311G(d,p) level of theory: χ = −(E_HOMO_ + E_LUMO_)/2 (electronegativity), Pi = −χ (chemical potential), η = (E_LUMO_-E_HOMO_)/2 (chemical hardness), σ = 1/η (chemical softness), and ω = Pi^2^/η (electrophilicity index). Data are presented in eV.

Compound	χ	Pi	η	σ	ω
**3**	3.31	−3.31	2.31	0.43	2.37
**4a**	3.88	−3.88	2.33	0.43	3.23
**4b**	3.92	−3.92	2.39	0.42	3.21
**4c**	4.62	−4.62	1.83	0.55	5.83
**4d**	3.83	−3.83	2.43	0.41	3.02

**Table 6:** Lowest estimated Gibbs energies of interaction in kcal mol^−1^ between the (*S*)-enantiomers of the compounds **3** to **4d** from the series and the muscarinic receptors MR2 and MR3 and the interleukin-β (IL-β) obtained through molecular docking via AutoDock 4.2.

Compound	MR2	MR3	IL-β
**3**	−9.9	−9.5	−10.5
**4a**	−11.7	−9.8	−11.1
**4b**	−12.0	−9.9	−12.0
**4c**	−11.8	−10.7	−12.2
**4d**	−11.4	−9.3	−12.2

**Table 7:** Relative change (%) in the parameters of spontaneous contractile activity of gastric smooth muscles under the action of drug agents.

	3		4a		4b		4c		4d		Mebeverine		Diclofenac	
Muscle tonus	10 *	↓	30 *	↓	54 *	↓	27 *	↓	25 *	↓	17 *	↓	16 *	↓
Amplitude of contraction	78 *	↓	20 *	↓	11	↓	67 *	↓	2	↓	33 *	↓	9	↓
Frequency of rhythmic contractions	1	↓	12	↓	11	↑	26 *	↑	9	↓	2	↓	3	↓
Tonic part of ACh reaction	55 *	↓	49 *	↓	59 *	↓	33 *	↓	43 *	↓	100 *	↓	38 *	↓

Base activity (tonic, phasic, amplitude) is taken as 100%; * indicates the values that differ significantly (*p* < 0.05) compared to the respective control parameters; ↓ indicates decreasing change; ↑ indicates incremental change.

**Table 8:** IC50 values of compounds **3**, **4a**–**d**.

Compound	Diclofenac	ASA	Mebeverine	3	4a	4b	4c	4d
IC50	17.4	11.2	0.91	0.76	0.87	0.93	0.94	0.83

**Table 9:** Immunohistochemical response for mebeverine derivatives, compared to mebeverine and diclofenac.

Compound	Diclofenac	Mebeverine	3	4a	4b	4c	4d
nNOS	172.15	117.55	167.18	175.48	143.82	116.35	135.12
IL-1β	165.85	175.48	160.52	150.12	164.32	121.02	177.52

## Data Availability

The original contributions presented in the study are included in the article/Supplementary Materials, further inquiries can be directed to the corresponding author.

## Supplementary Materials

The following supporting information can be downloaded at: https://www.mdpi.com/article/10.3390/biomedicines12102321/s1, Figure S1: ^1^H-NMR spectrum of compound **3**, Figure S2: ^13^C-NMR spectrum of compound **3**, Figure S3: FT-IR spectrum of compound **3**, Figure S4: Mass spectrum of **3**, Figure S5: ^1^H-NMR spectrum of compound **4a**, Figure S6: ^13^C-NMR spectrum of compound **4a**, Figure S7: FT-IR spectrum of compound **4a**, Figure S8: Mass spectrum of **4a**, Figure S9: ^1^H-NMR spectrum of compound **4b**, Figure S10: ^13^C-NMR spectrum of compound **4b**, Figure S11: FT-IR spectrum of compound **4b**, Figure S12: Mass spectrum of **4b**, Figure S13: ^1^H-NMR spectrum of compound **4c**, Figure S14: ^13^C-NMR spectrum of compound **4c**, Figure S15: FT-IR spectrum of compound **4c**, Figure S16: Mass spectrum of **4c**, Figure S17: ^1^H-NMR spectrum of compound **4d**, Figure S18: ^13^C-NMR spectrum of compound **4d**, Figure S19: FT-IR spectrum of compound **4d**, Figure S20: Mass spectrum of **4d**, Table S1: Lowest estimated Gibbs energies of interaction in kcal mol−1 between the (R)-isomers of **3**, **4a**–**d** and the two ligand-binding centers of human serum albumin (HSA) known as Sudlow 1 and Sudlow 2, (HSA Sud1 and HSA Sud2), the muscarinic receptors MR2 and MR3, and the interleukin-β (IL-β) obtained through molecular docking via AutoDock 4.2, Figure S21: Optimized geometries and obtained energy differences between conformers of (S)-isomer of compound **3** (in kcal mol−1), Figure S22: B3LYP/6-311G(d,p) optimized structures of the (R)-isomers of the series of compounds, Figure S23: The highest occupied and lowest unoccupied molecular orbitals (HOMO/LUMO) for the (R)-isomers of the series.
