# Supplementary material for: Spasmolytic Activity and Anti-Inflammatory Effect of Novel Mebeverine Derivatives
Source: Biomedicines. 2024 Oct 12;12(10):2321. doi: 10.3390/biomedicines12102321 (PMC11505310; doi:10.3390/biomedicines12102321)

Spasmolytic activity and anti-inflammatory effect of novel mebeverine derivatives

Mihaela Stoyanova, Miglena Milusheva, Vera Gledacheva, Iliyana Stefanova, Mina Todorova, Mina Pencheva, Kirila Stojnova, Slava Tsoneva, and Stoyanka Nikolova

Table of Contents:

Figure S1: ^{1}H-NMR spectrum of compound **3**, page 3<br>Figure S2: ^{13}C-NMR spectrum of compound **3**, page 4<br>Figure S3: FT-IR spectrum of compound **3**, page 5<br>Figure S4: Mass spectrum of **3**, page 6	Figure S5: ^{1}H-NMR spectrum of compound **4a**, page 7<br>Figure S6: ^{13}C-NMR spectrum of compound **4a**, page 8<br>Figure S7: FT-IR spectrum of compound **4a**, page 9<br>Figure S8: Mass spectrum of **4a**, page 10
Figure S9: ^{1}H-NMR spectrum of compound **4b**, page 11<br>Figure S10: ^{13}C-NMR spectrum of compound **4b**, page 12<br>Figure S11: FT-IR spectrum of compound **4b**, page 13<br>Figure S12: Mass spectrum of **4b**, page 14	Figure S13: ^{1}H-NMR spectrum of compound **4c**, page 15<br>Figure S14: ^{13}C-NMR spectrum of compound **4c**, page 16<br>Figure S15: FT-IR spectrum of compound **4c**, page 17<br>Figure S16: Mass spectrum of **4c**, page 18
Figure S17: ^{1}H-NMR spectrum of compound **4d**, page 19	Table S1. Lowest estimated Gibbs energies of interaction in kcal mol-1 between the (R)-isomers of **3**, **4a-d** and the two ligand-binding centers of human serum albumin (HSA) known as Sudlow 1 and Sudlow 2, (HSA Sud1 and HSA Sud2), the muscarinic receptors MR2 and MR3, and the interleukin-β IL-β obtained through molecular docking via AutoDock 4.2., page 23
Figure S18: ^{13}C-NMR spectrum of compound **4d**, page 20	Figure S21. Optimized geometries and obtained energy differences between conformers of (S)-isomer of compound **3** (in kcal mol-1), page 23

Figure S19: FT-IR spectrum of compound **4d**, page 21	Figure S22. B3LYP/6-311G(d,p) optimized structures of the (R)-isomers of the series of compounds, page 24
Figure S20: Mass spectrum of **4d**, page 22	Figure S23. The highest occupied and lowest unoccupied molecular orbitals (HOMO/LUMO) for the (R)-isomers of the series, page 25

Figure S1: ^{1}H-NMR spectrum of compound 3

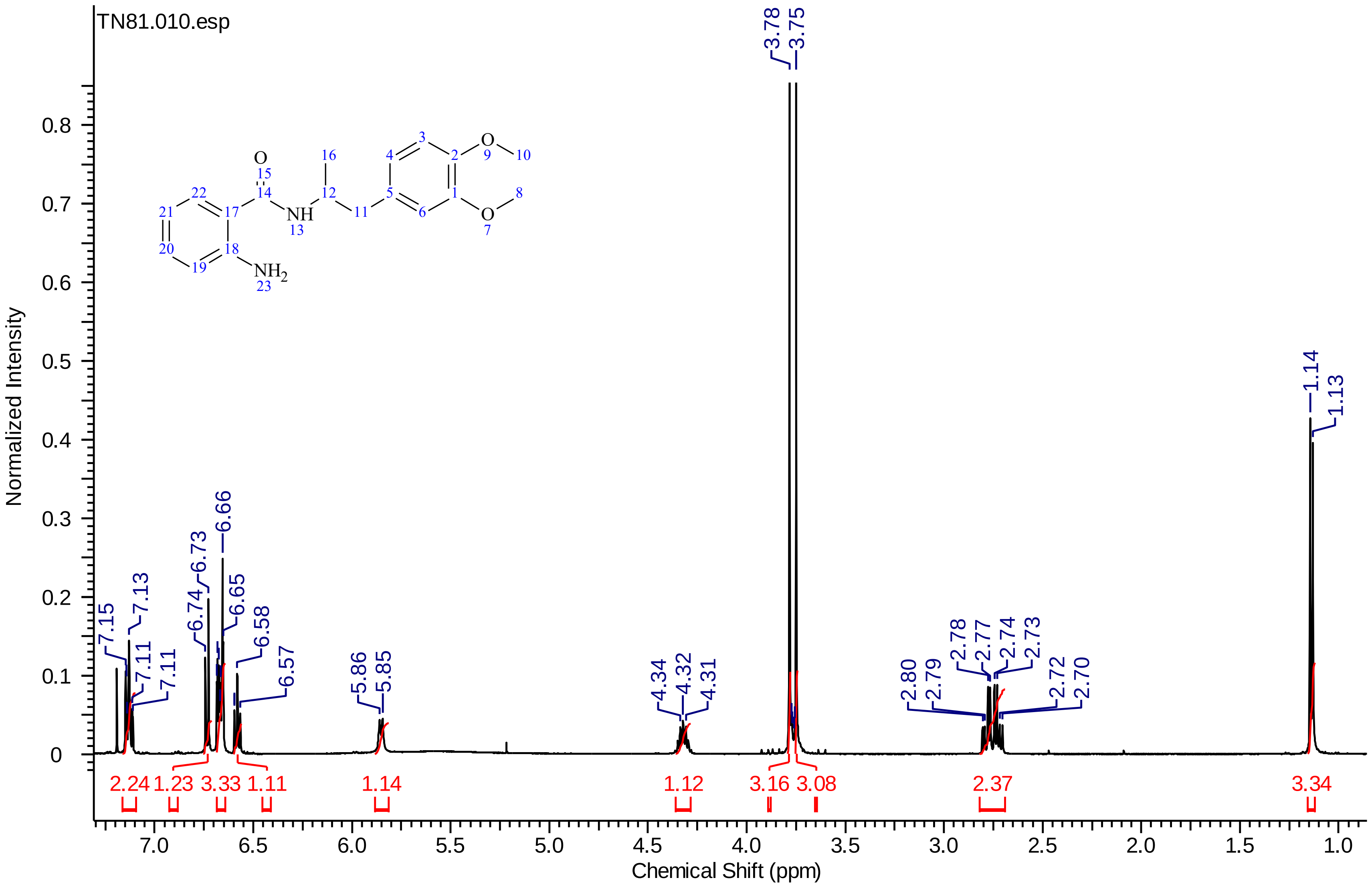

Figure S2: ^{13}C-NMR spectrum of compound **3**

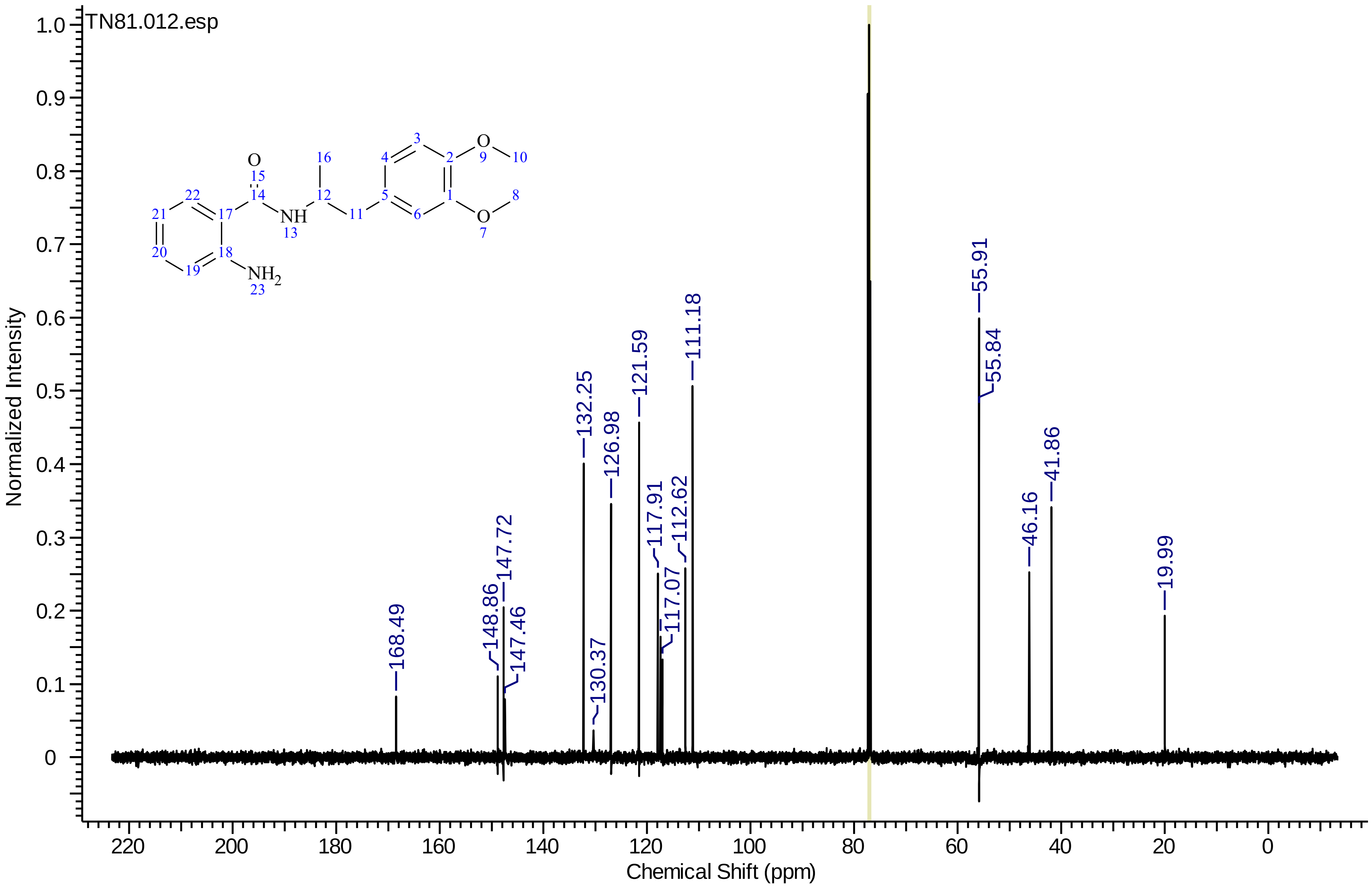

Figure S3: FT-IR spectrum of compound **3**

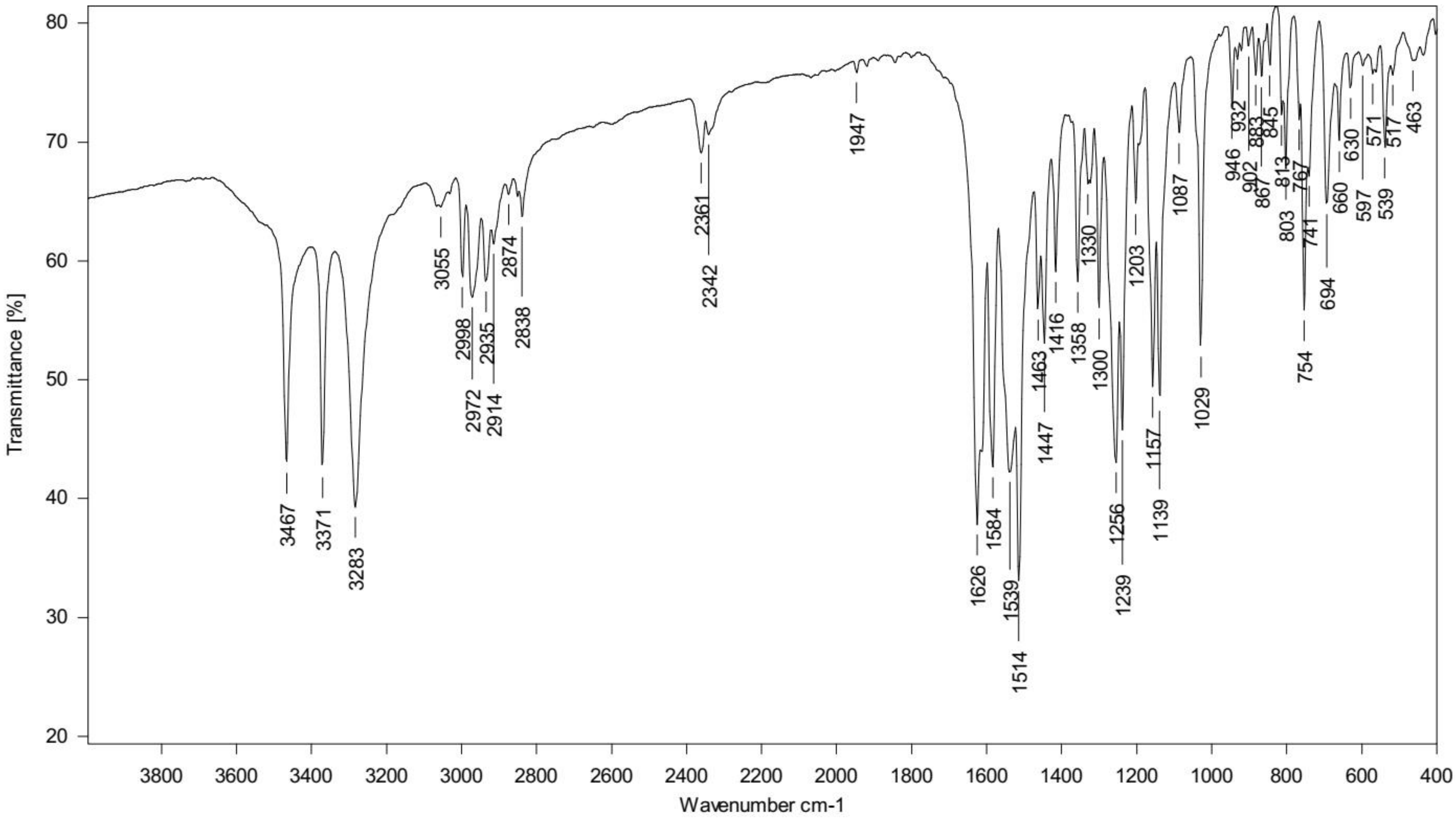

Figure S4: Mass spectrum of **3**

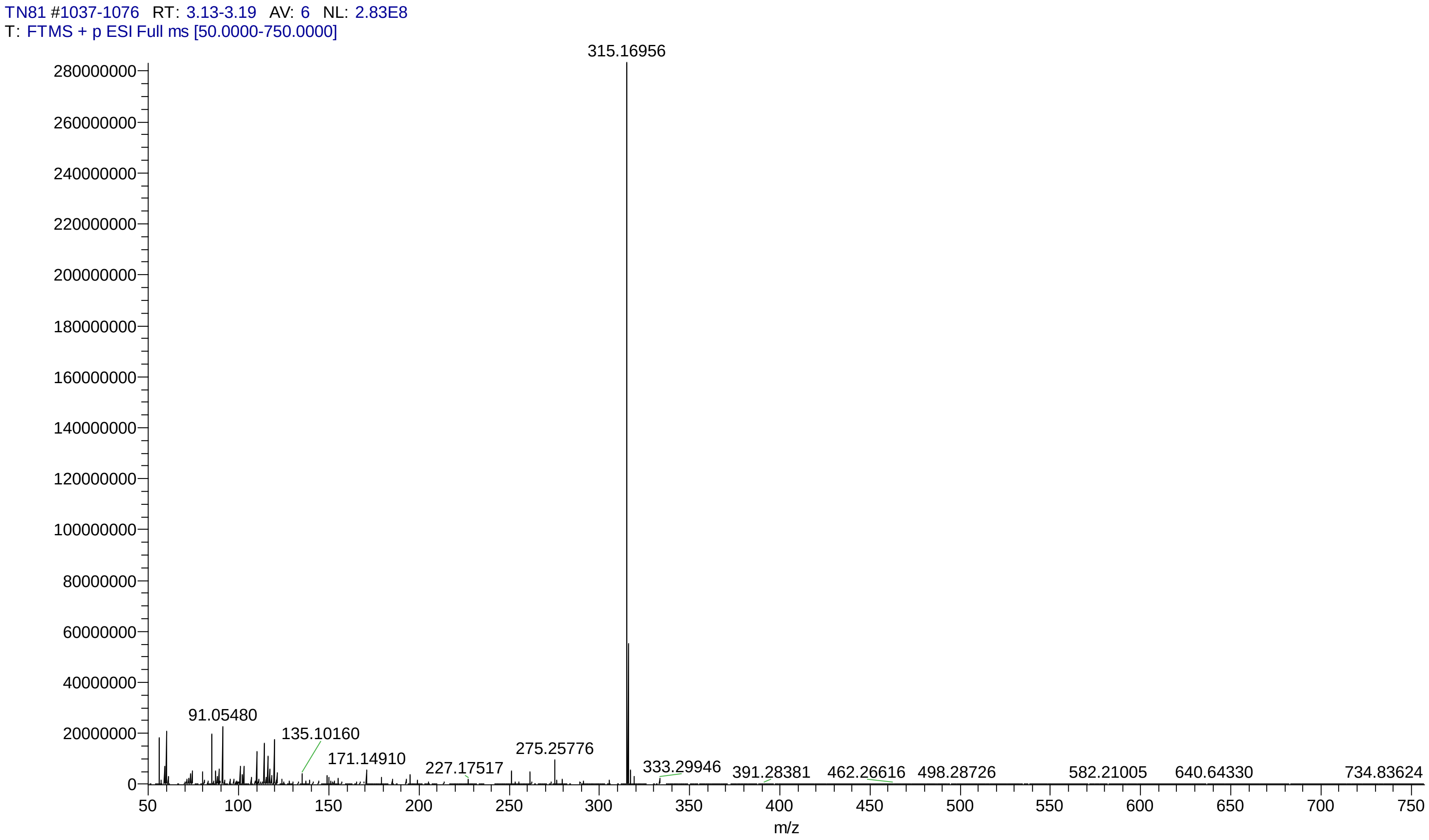

Figure S5: 1H-NMR spectrum of compound **4a**

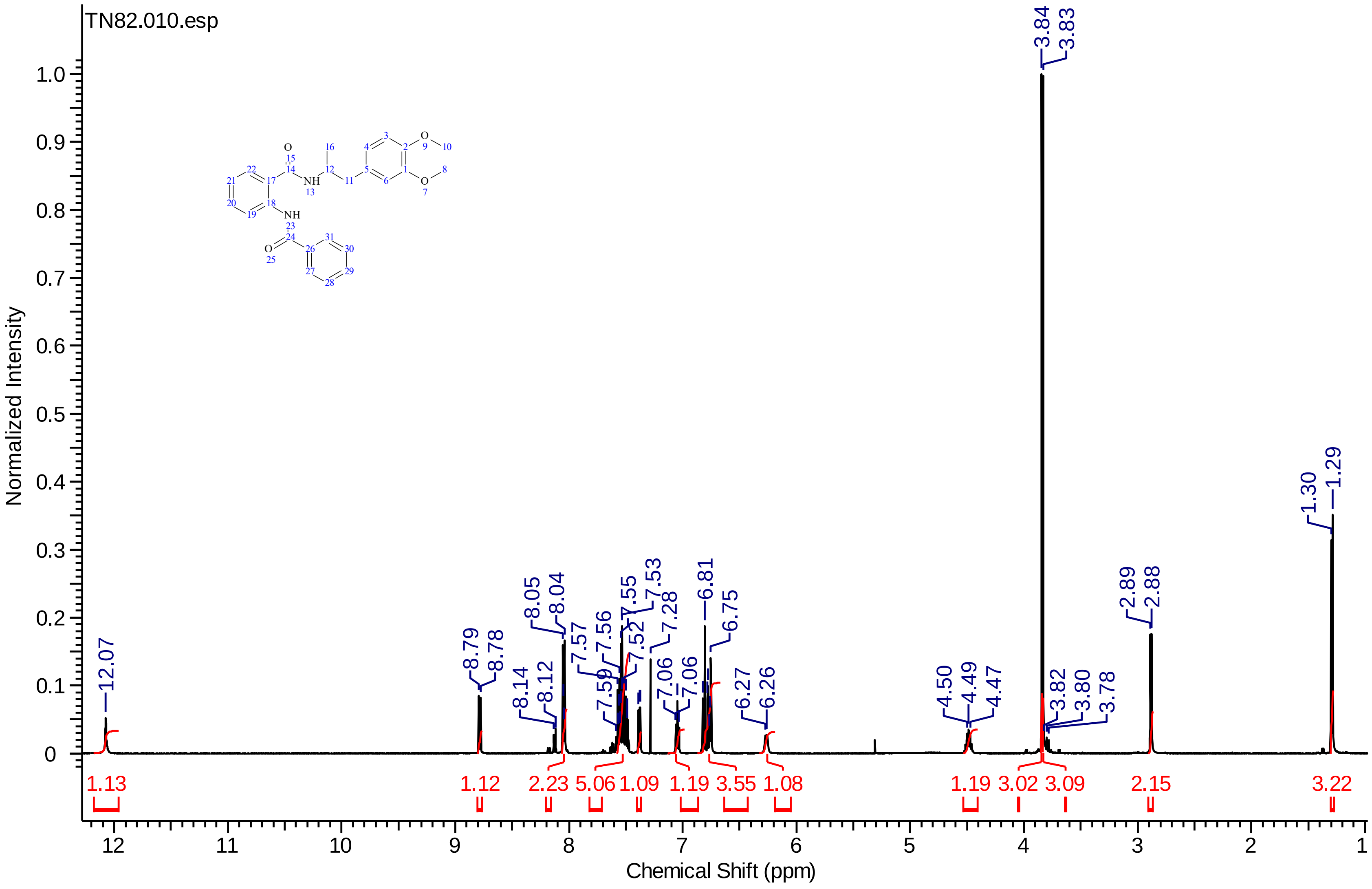

Figure S6: 13C-NMR spectrum of compound **4a**

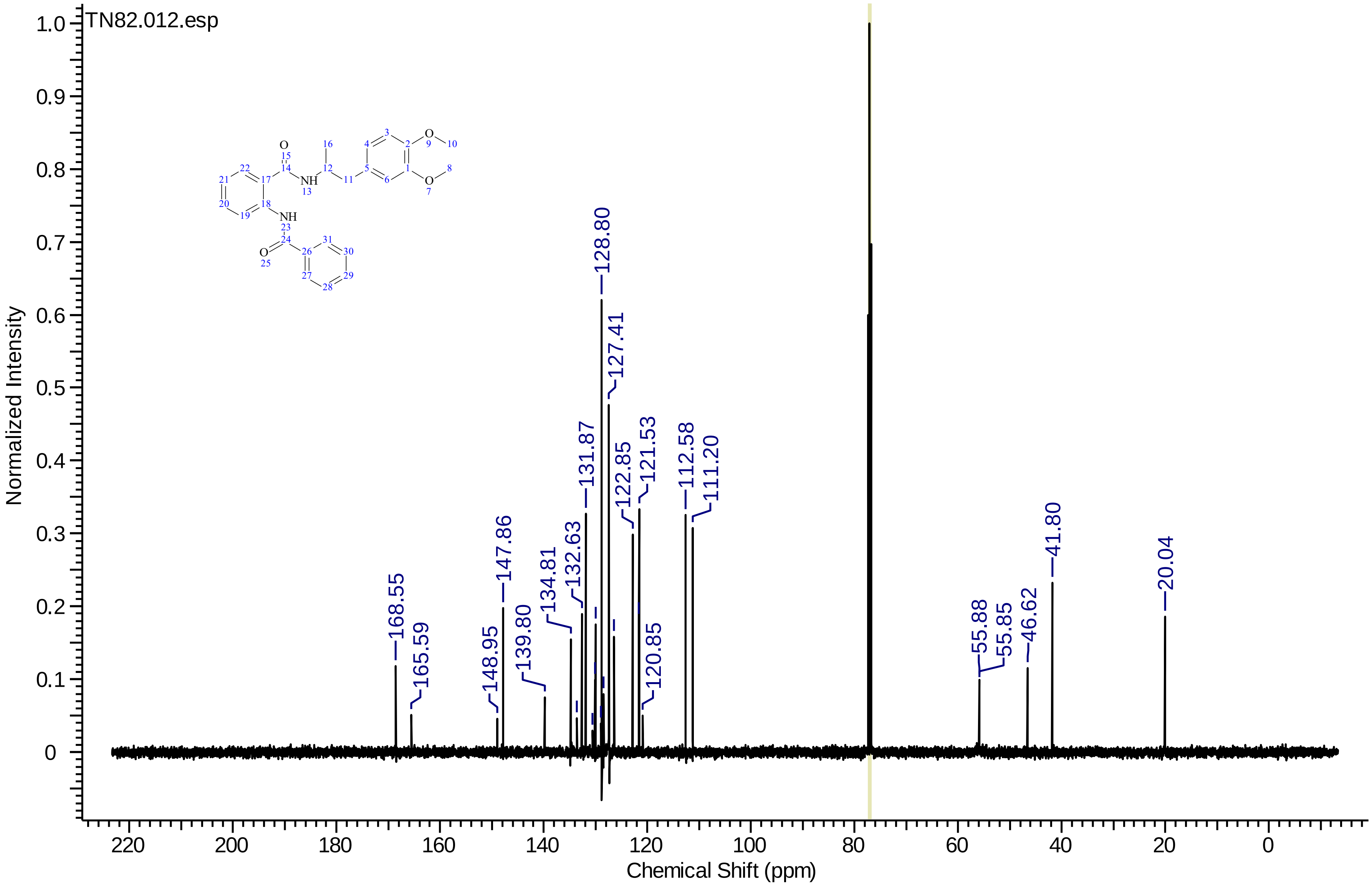

Figure S7: FT-IR spectrum of compound **4a**

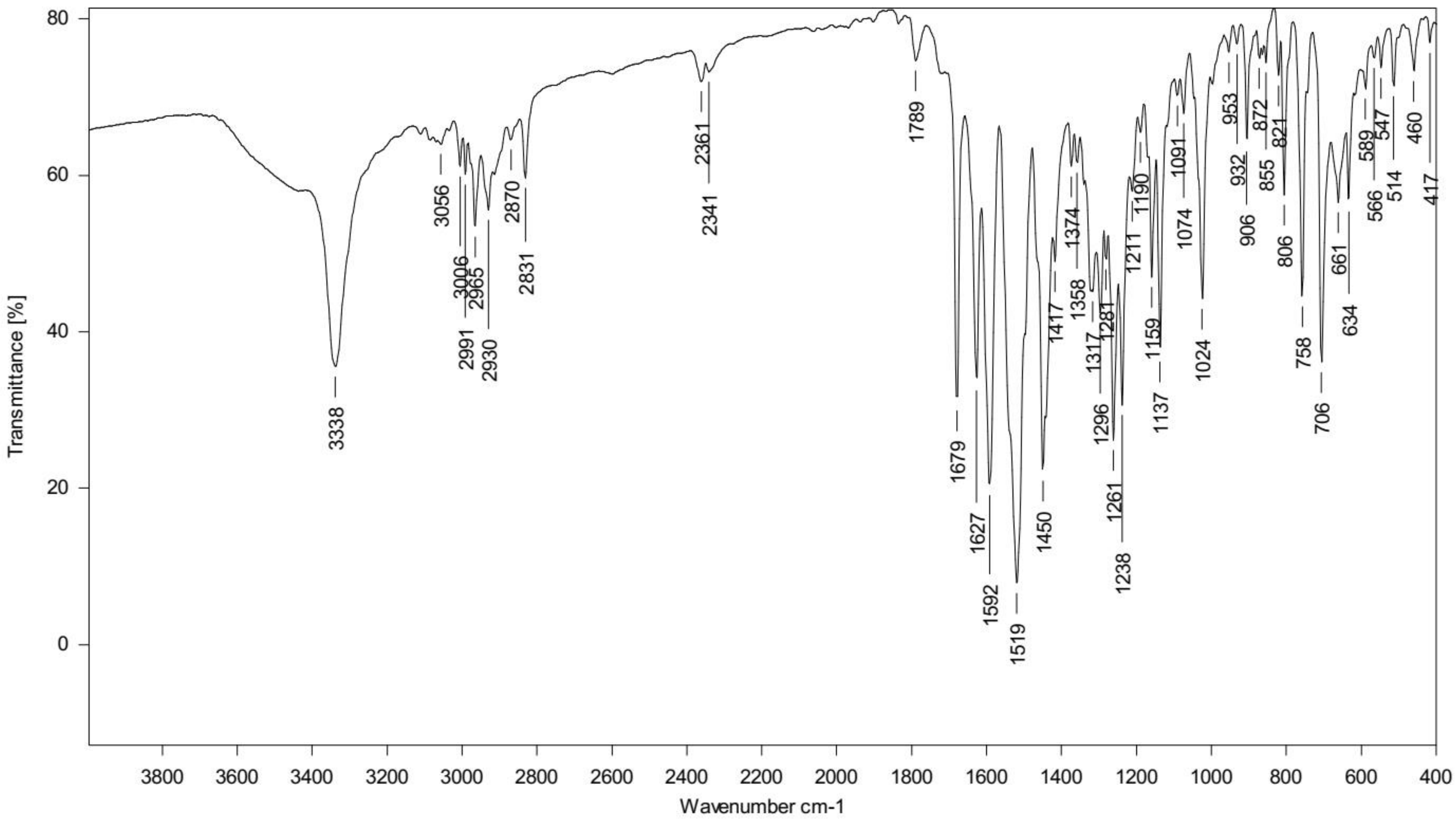

Figure S8: Mass spectrum of **4a**

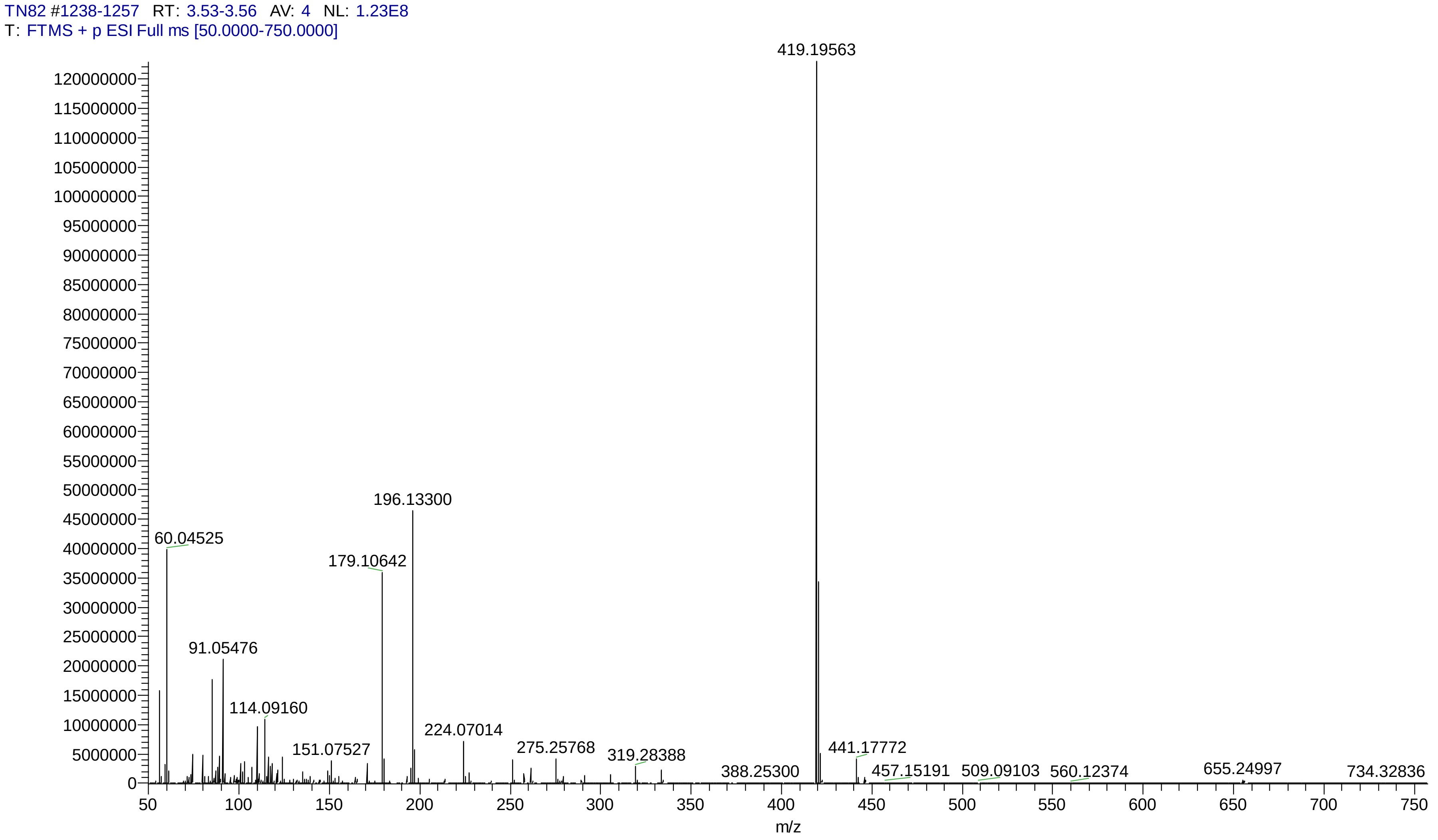

Figure S9: 1H-NMR spectrum of compound **4b**

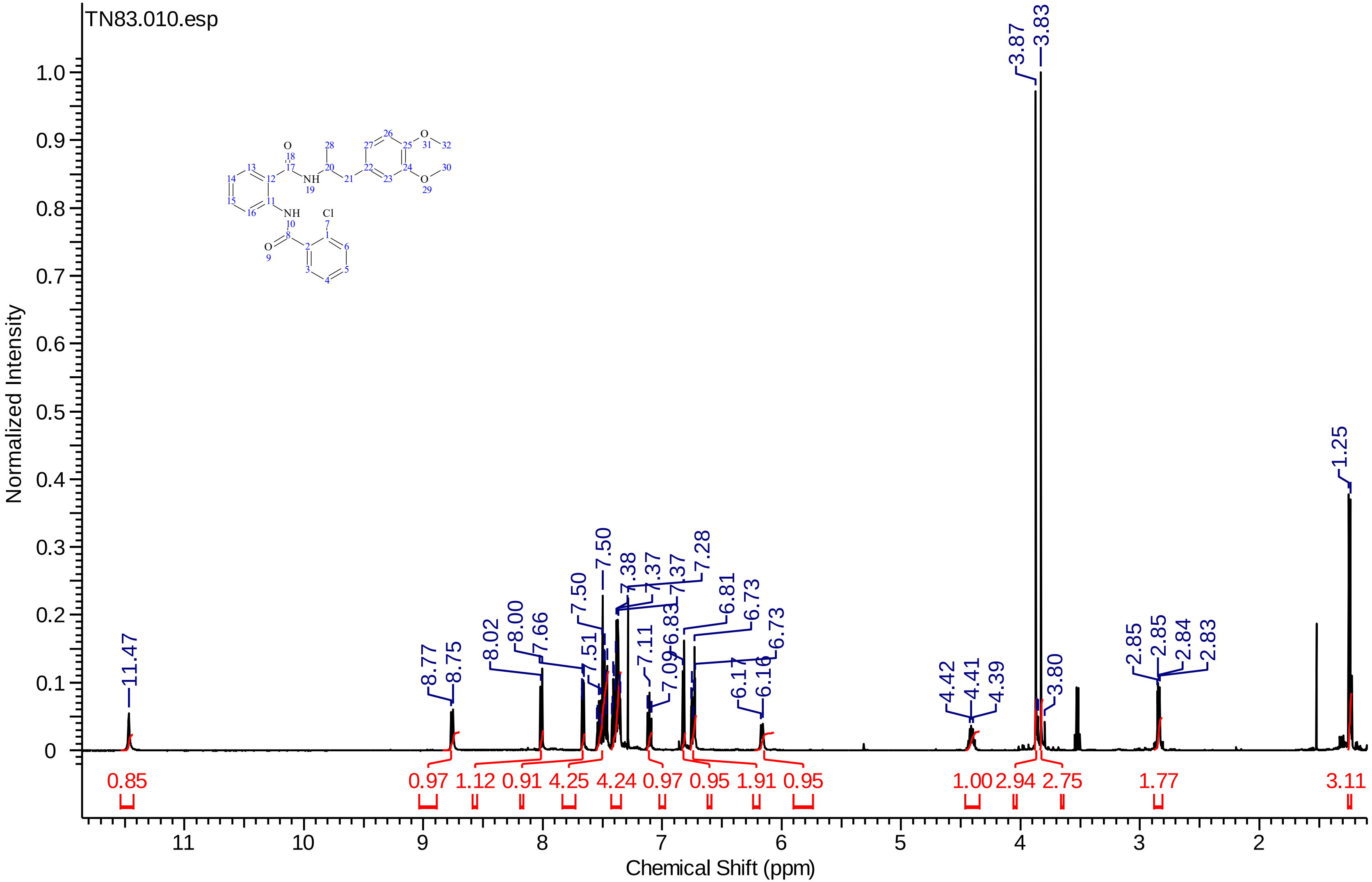

Figure S10: 13C-NMR spectrum of compound **4b**

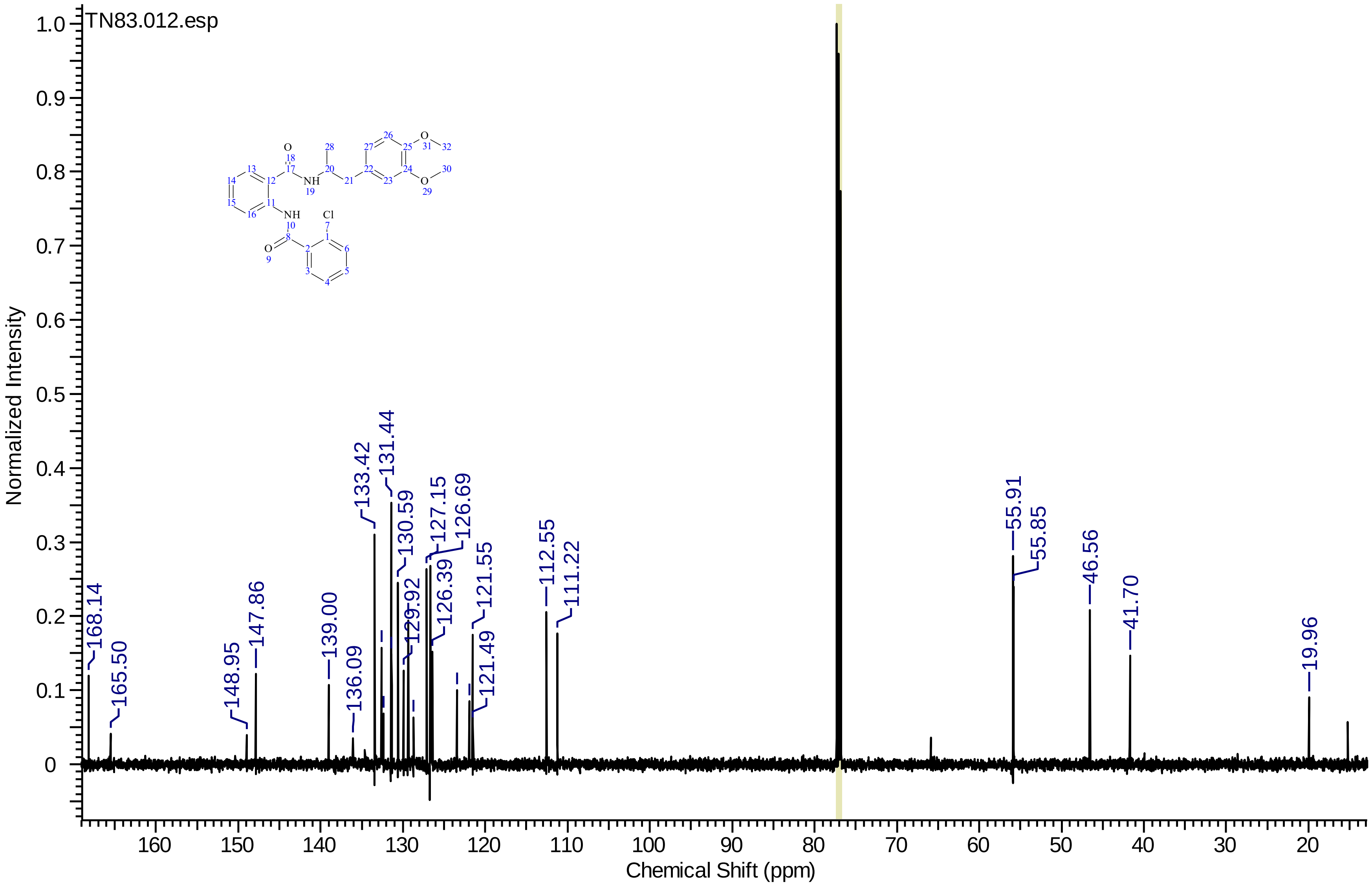

Figure S11: FT-IR spectrum of compound **4b**

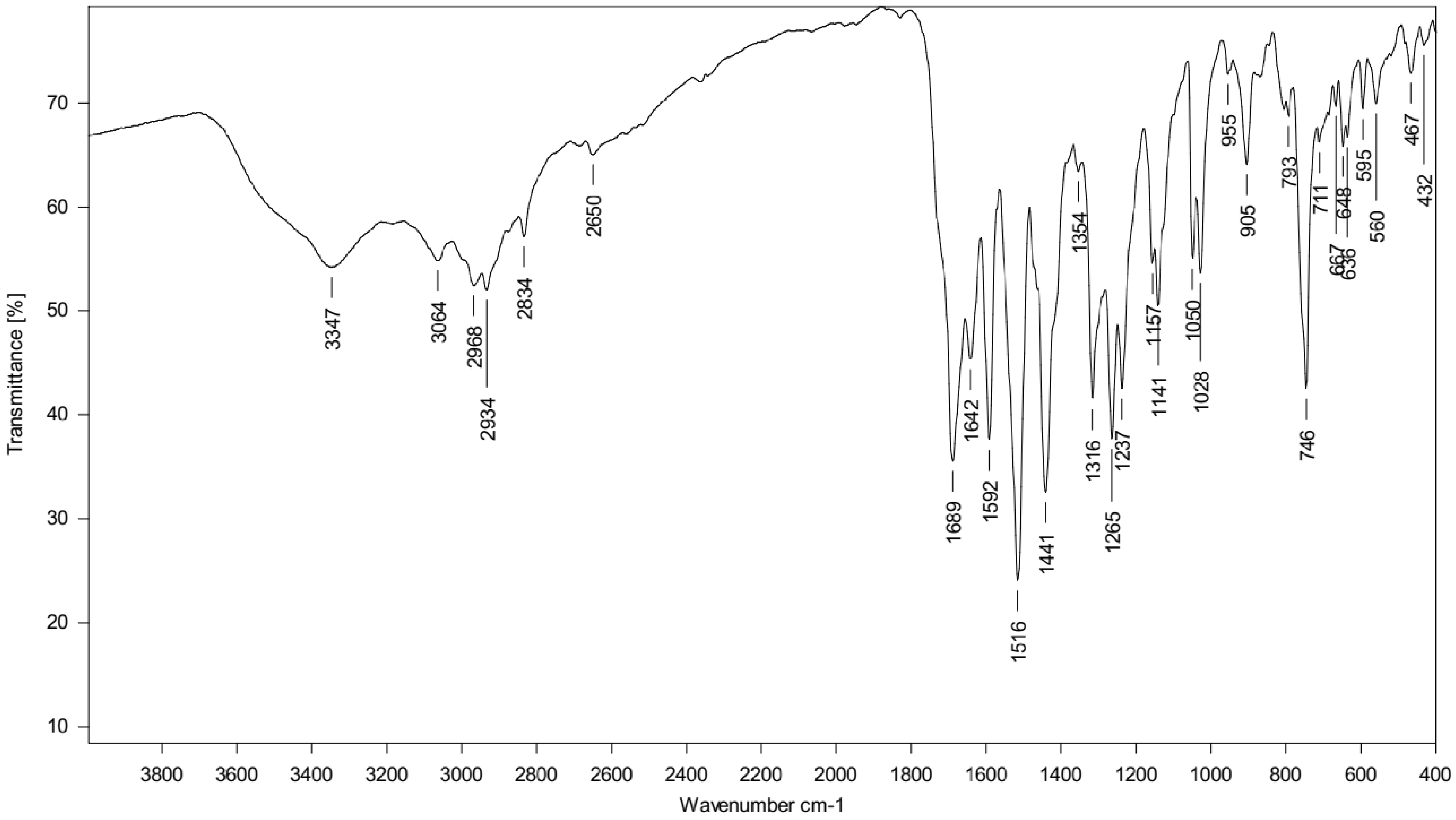

Figure S12: Mass spectrum of **4b**

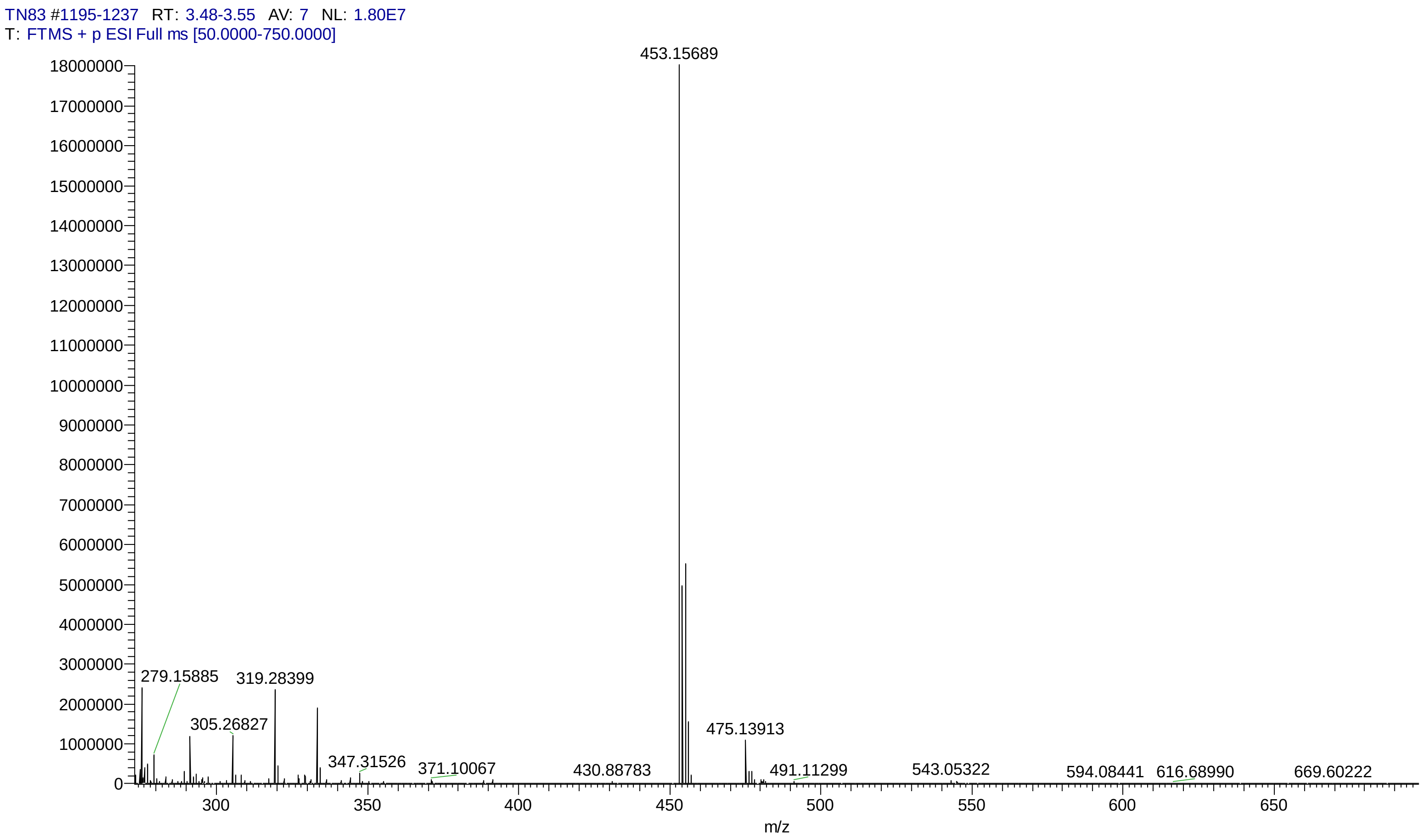

Figure S13: 1H-NMR spectrum of compound **4c**

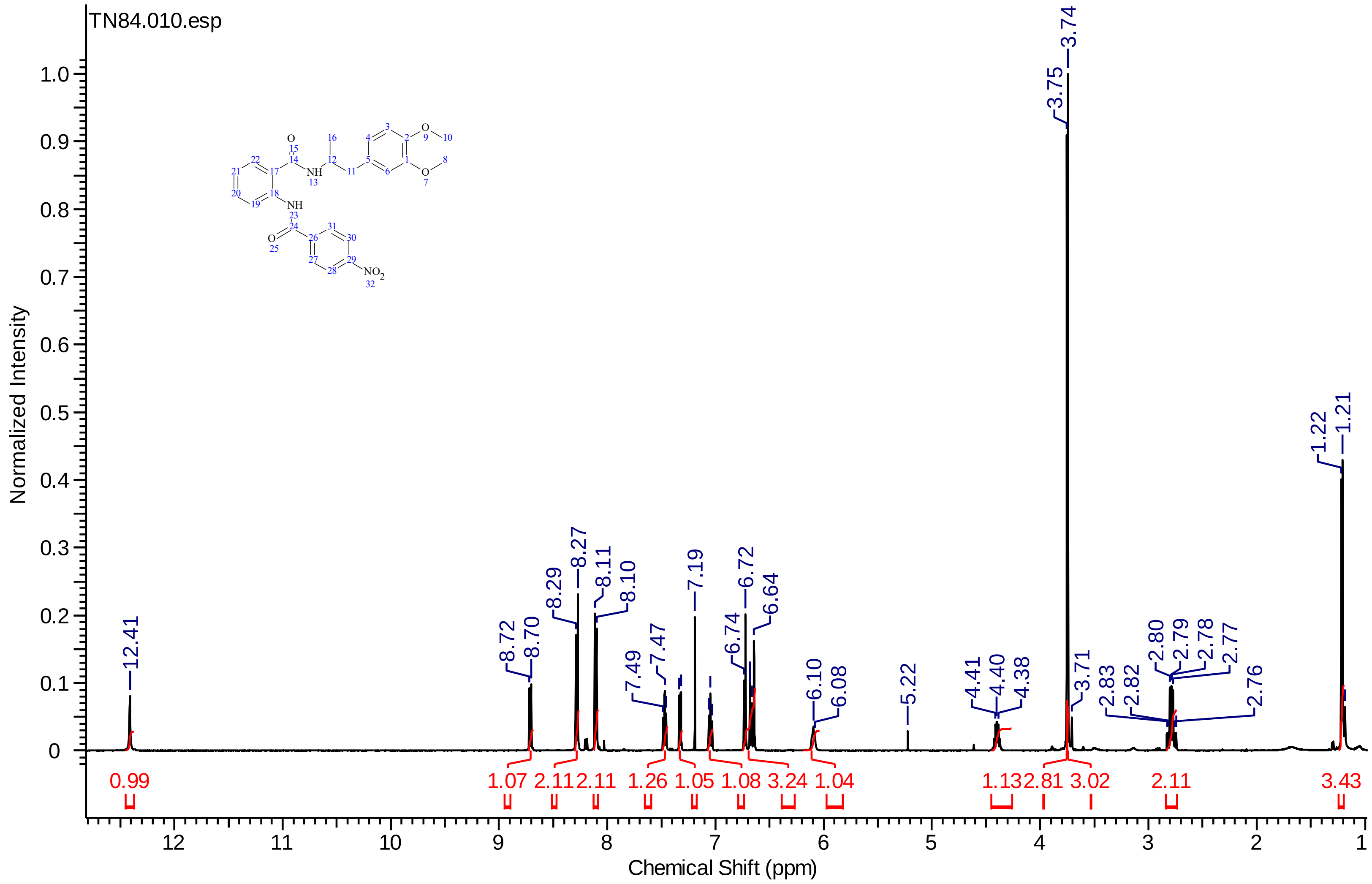

Figure S14: 13C-NMR spectrum of compound **4c**

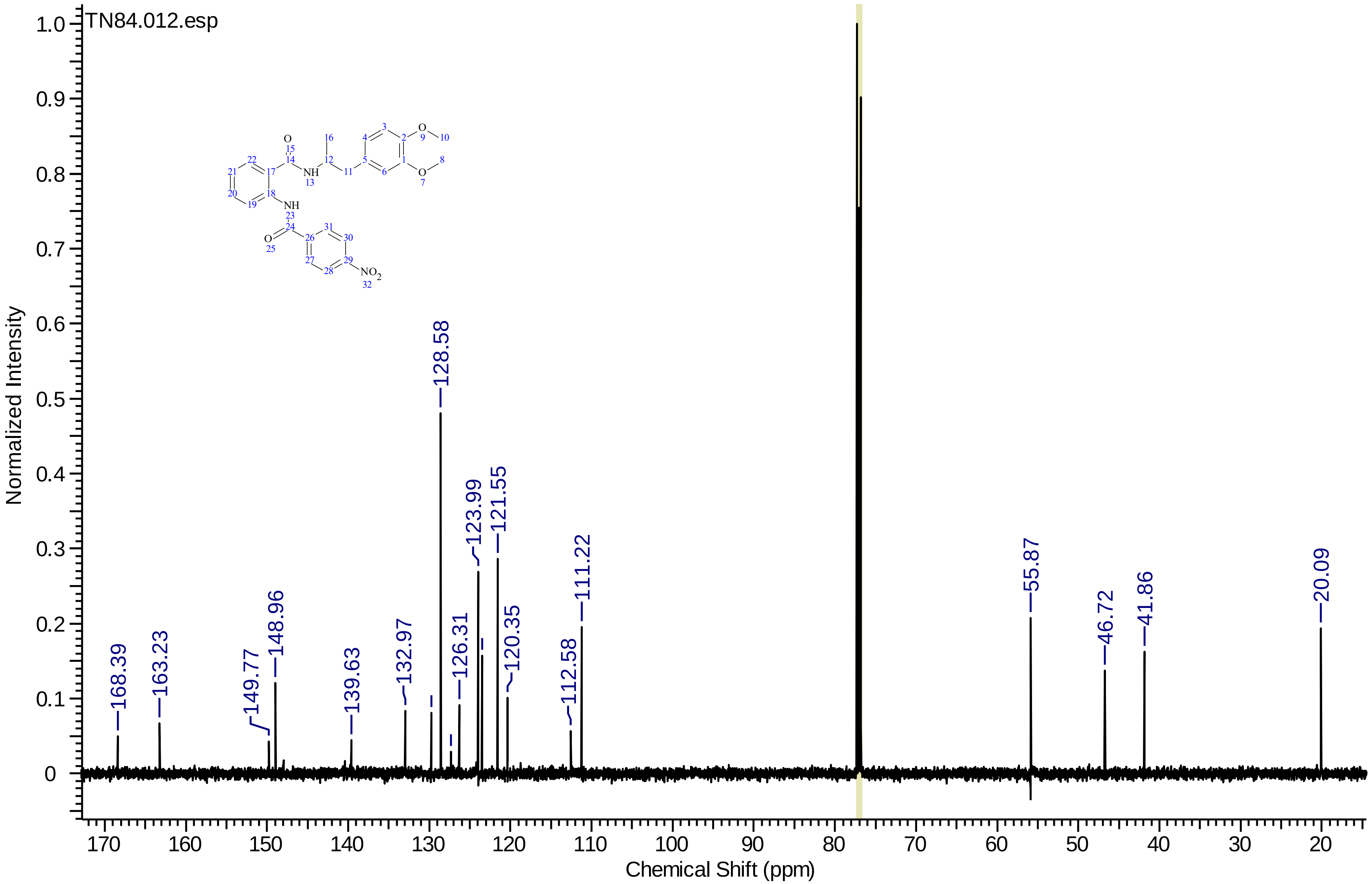

Figure S15: FT-IR spectrum of compound **4c**

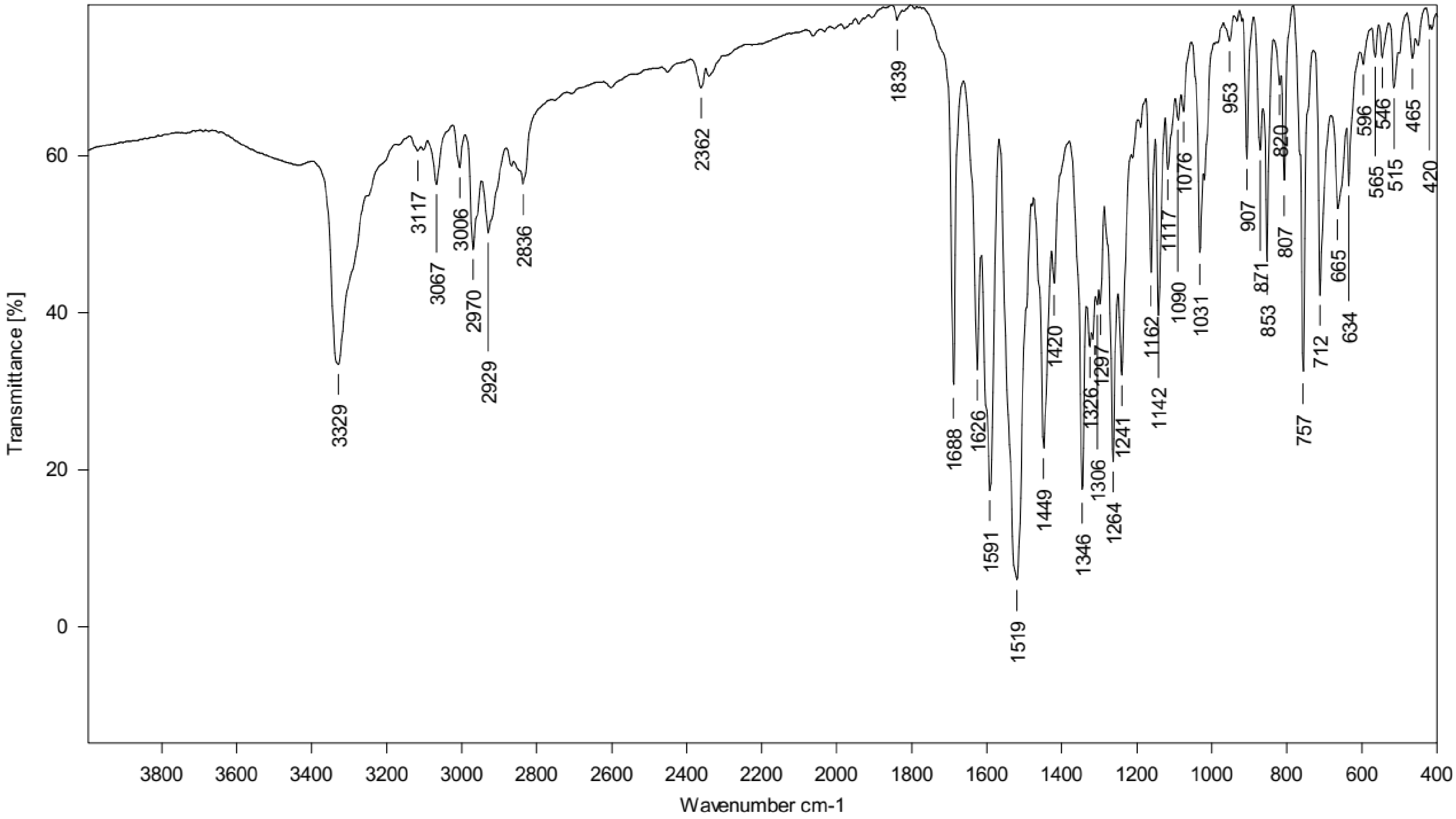

Figure S16: Mass spectrum of **4c**

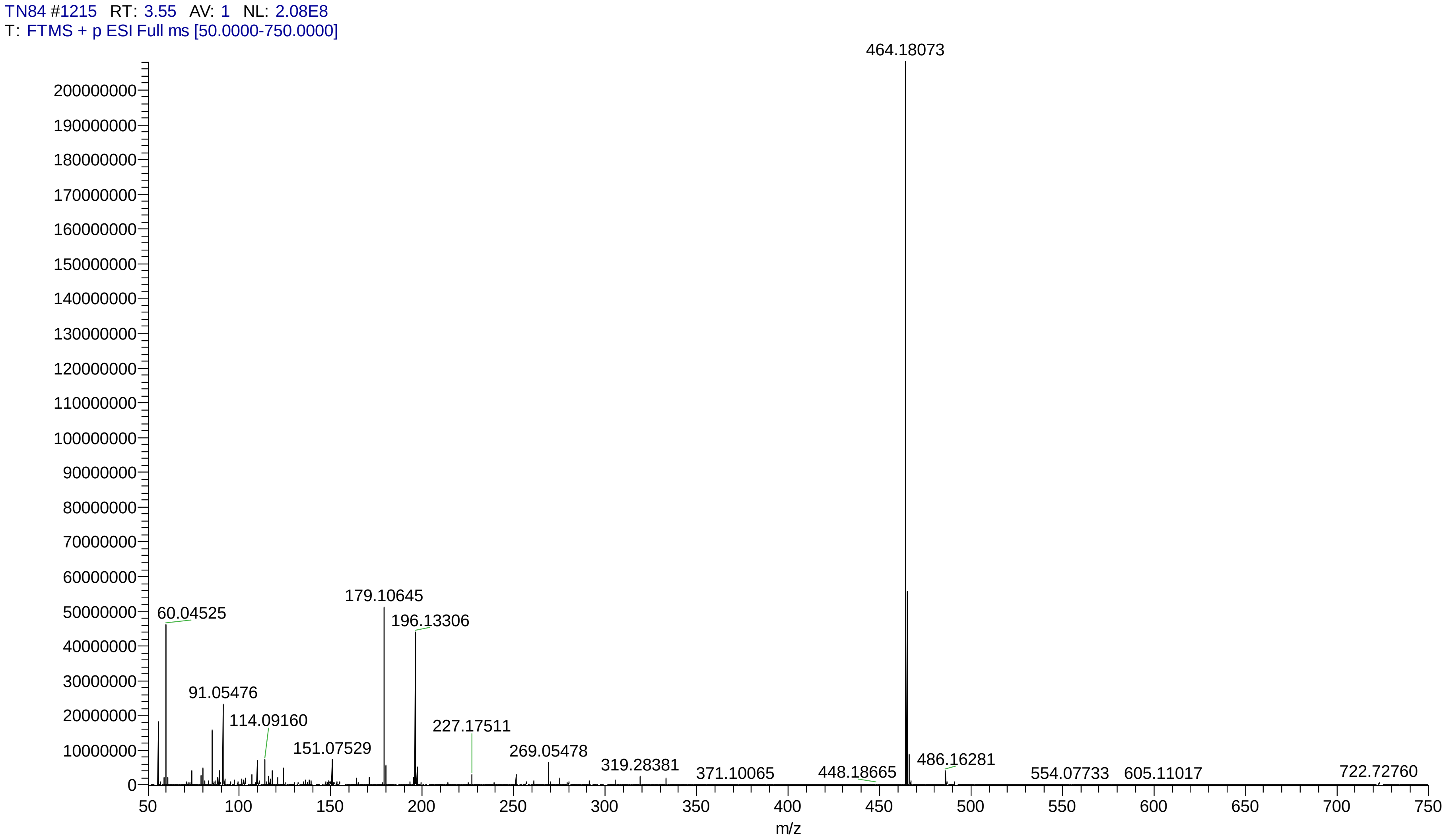

Figure S17: 1H-NMR spectrum of compound **4d**

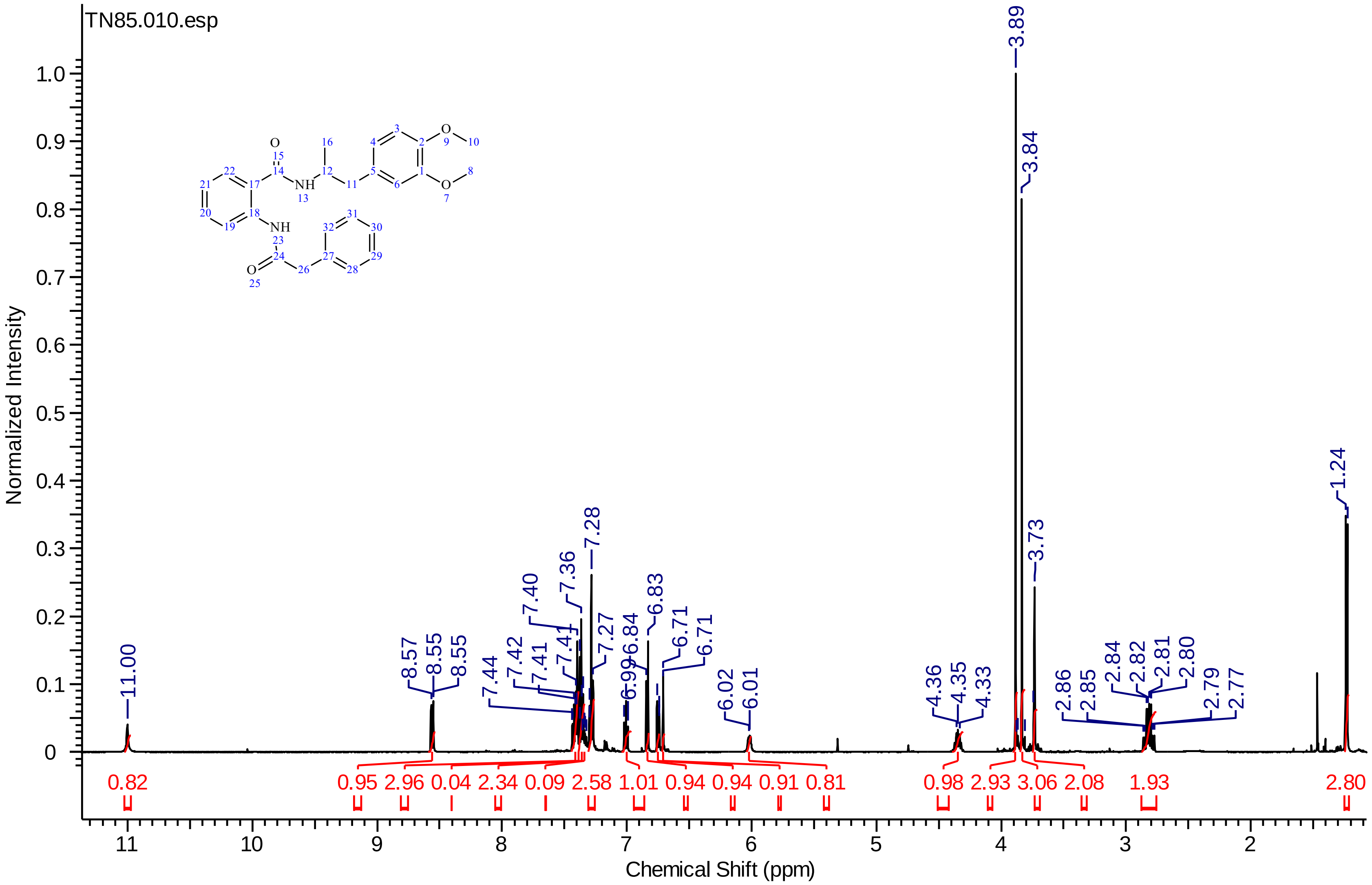

Figure S18: 13C-NMR spectrum of compound **4d**

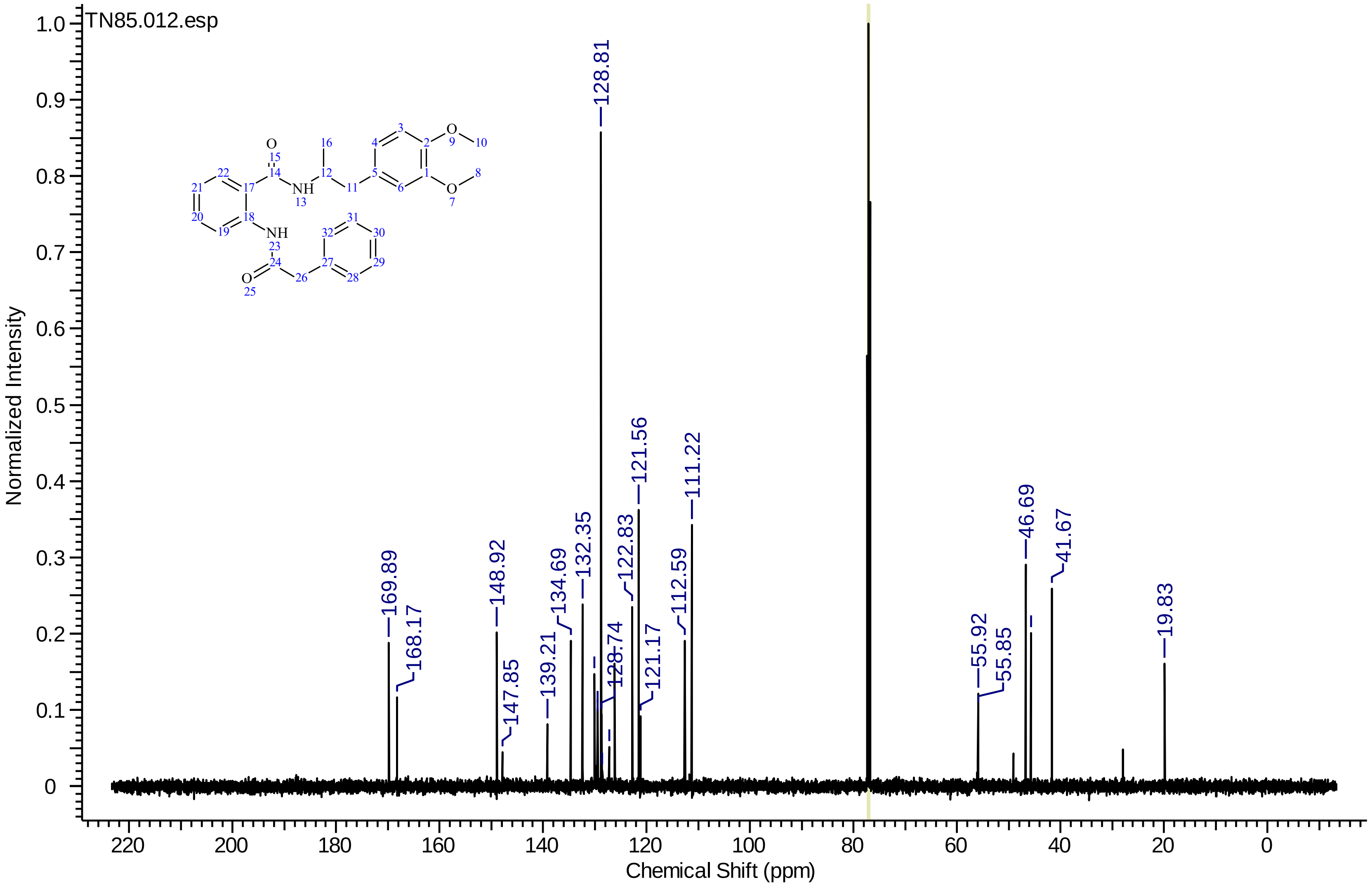

Figure S19: FT-IR spectrum of compound **4d**

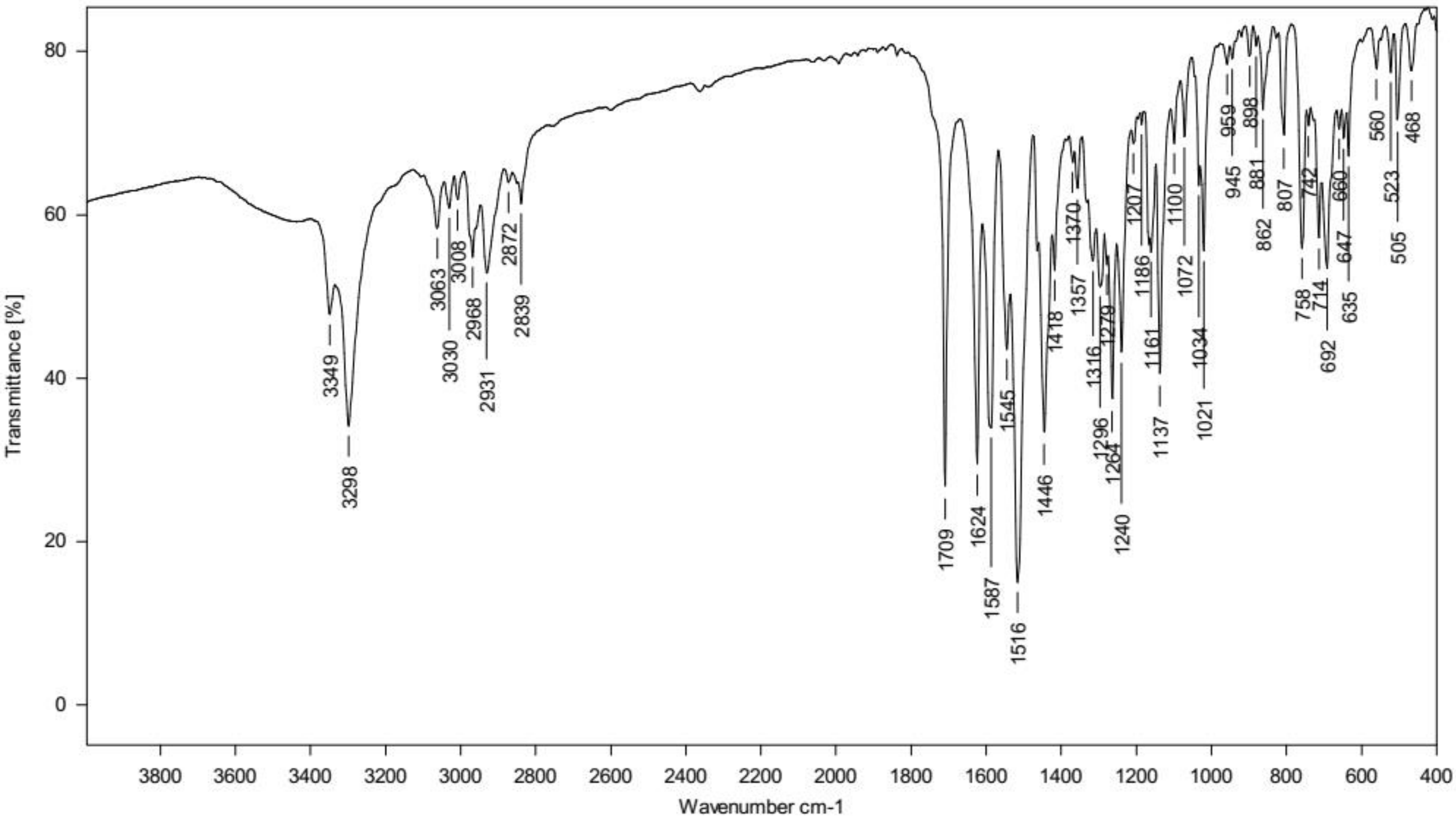

Figure S20: Mass spectrum of **4d**

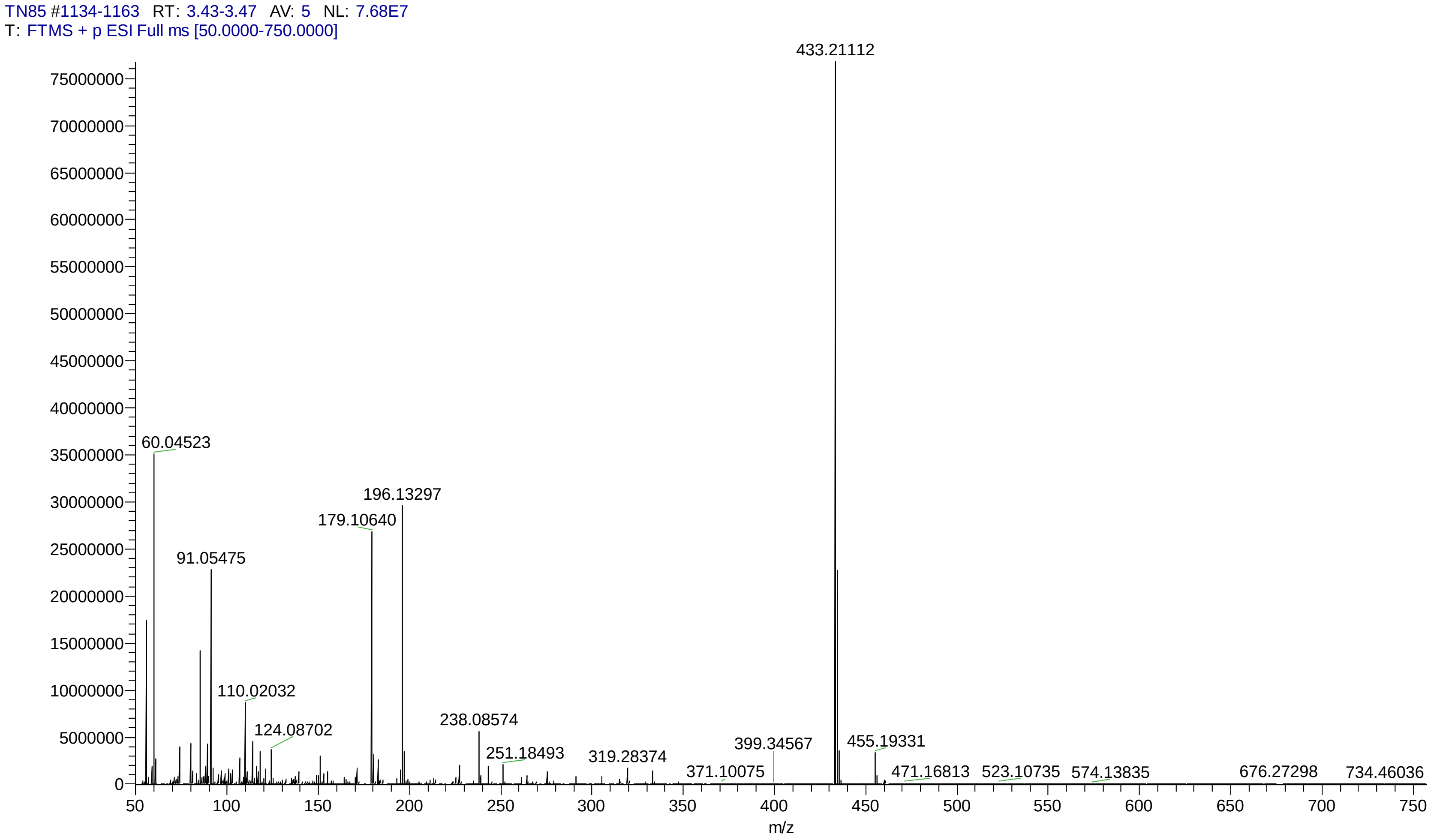

Table S1. Lowest estimated Gibbs energies of interaction in kcal mol^{-1} between the *(R)*-isomers of compounds from the series 3 to 4d and the two ligand-binding centers of human serum albumin (HSA) known as Sudlow 1 and Sudlow 2, (HSA Sud1 and HSA Sud2), the muscarinic receptors MR2 and MR3, and the interleukin-β IL-β obtained through molecular docking via AutoDock 4.2.

**Compound**	**HSA Sud 1**	**HSA Sud 2**	**MR2**	**MR3**	**IL-β**
**3**	-9.5	-8.3	-9.9	-9.5	-10.5
**4a**	-11.6	-8.3	-11.7	-9.8	-11.1
**4b**	-12.1	-8.6	-12.0	-9.9	-12.0
**4c**	-12.5	-9.7	-11.8	-10.7	-12.2
**4d**	-11.4	-8.6	-11.4	-9.3	-12.2

Figure S21. Optimized geometries and obtained energy differences between conformers of *(S)*-isomer of compound 3 (in kcal mol^{-1}).

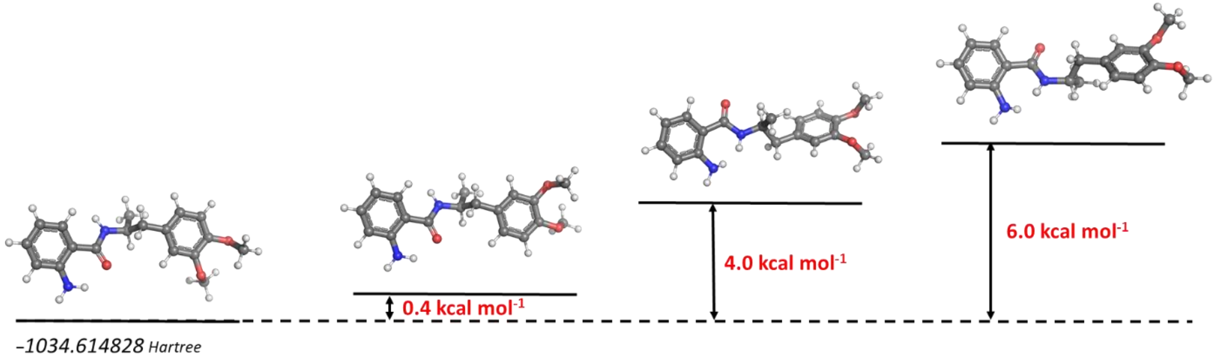

Figure S22. B3LYP/6-311G(d,p) optimized structures of the *(R)*-isomers of the series of compounds under study.

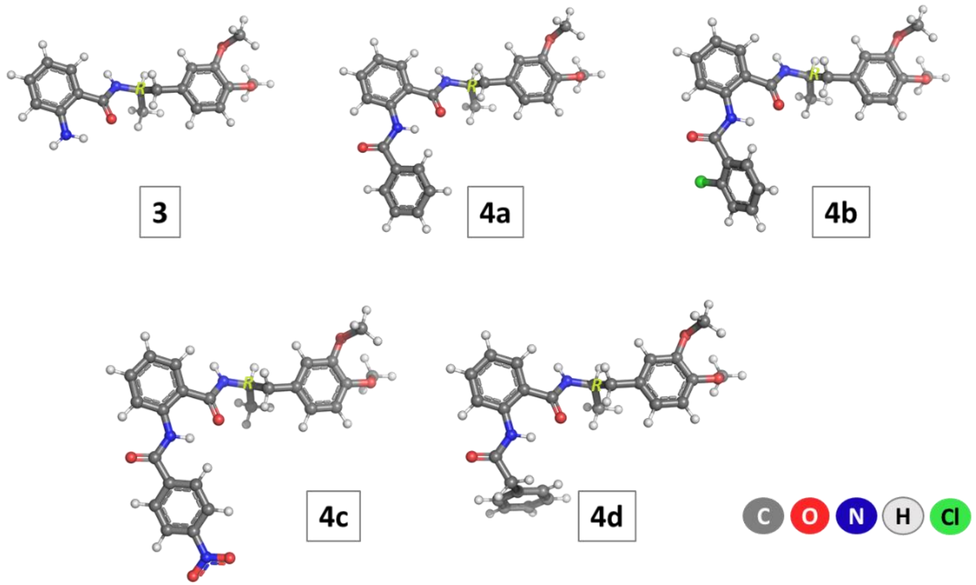

Figure S23. The highest occupied and lowest unoccupied molecular orbitals (HOMO/LUMO) for the *(R)*-isomers of the series.

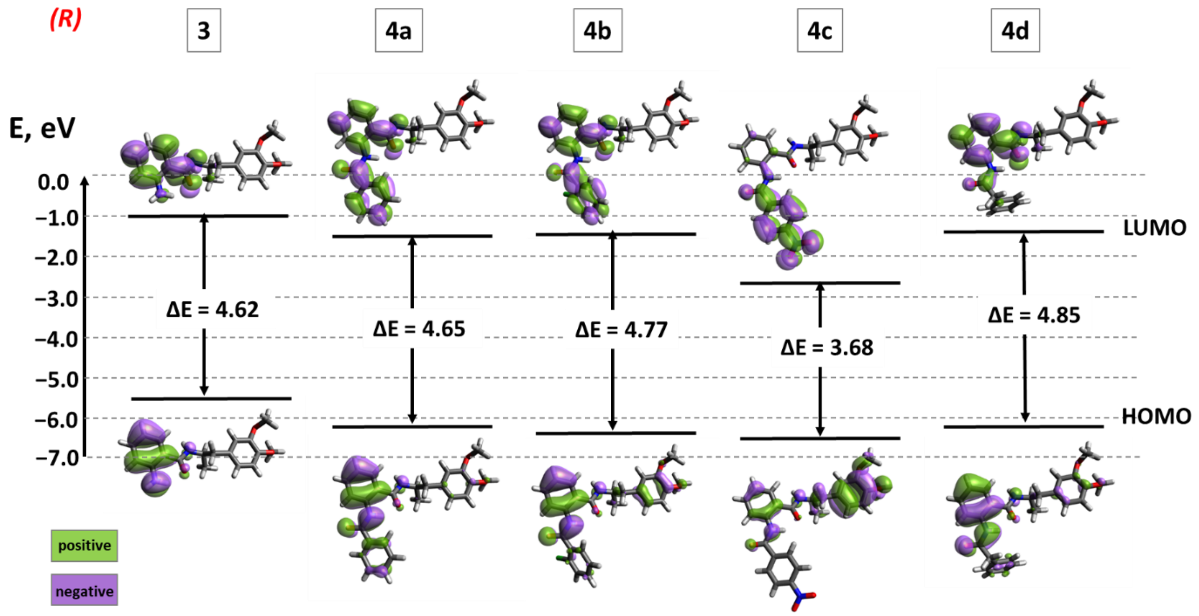

Supplement: Supplementary file 1 [file biomedicines-12-02321-s001.zip › biomedicines-3211523-supplementary.pdf]
